# Simulation analysis of non-respondent information in context of small domain

**DOI:** 10.1016/j.heliyon.2024.e26897

**Published:** 2024-03-01

**Authors:** Ashutosh Ashutosh, Marius Stefan, Piyush Kant Rai, Walid Emam, Soofia Iftikhar, Malik Muhammad Anas

**Affiliations:** a Department of Statistics, Allahabad Degree College, University of Allahabad, Prayagraj, 211003, India; b Department of Applied Mathematics, Faculty of Applied Sciences, Polytechnic University of Bucharest, Splaiul Independentei, nr. 313, RO-060042, USA; c Department of Statistics, Institute of Science, Banaras Hindu University, Varanasi, 221005, India; d Department of Statistics and Operations Research, Faculty of Science, King Saud University, P.O. Box 2455, Riyadh, 11451, Saudi Arabia; e Department of Statistics, Shaheed Benazir Bhutto Women University Peshawar, KP, Pakistan; f Department of Economics and Statistics, University of Salerno, Fisciano, Salerno, 84084, Italy

**Keywords:** Auxiliary variable, Calibration approach, Sample size, Simulation, Small domain, Unit non-response

## Abstract

In the real-world, there are various situations when all units are not accessible of the respondent called unit non-response. The effect of unit non-response is a tricky matter for estimating the total number of unit. The present work highlights the interest about subpopulations (domains) in two affairs: i. if domains total of the supportive information is accessible ii. if domains total of the supportive variable does not access. The government needs to be introducing the actual facilities in these small domains. The supportive information is used to find out the estimate of the non respondent information and to apply this information for desired domains. Sometimes, it has been found that the accessible auxiliary variable for the domains might be positive shape. Therefore, it develops an appropriate model that has positive skewness. The present context highlighted the indirect method using a power-based estimation with calibration approach. By combining power based estimation and calibration technique, it is possible to obtain more accurate estimates for intended small domains. Even the supportive information is positively biased. This approach helps us in mitigating the effect of non-respondent and improving the overall reliability of the estimators. The simulation was conducted for different sizes 70 and 90 when nonresponse variable in the study variable. The results show that investigated power-based estimate provides better option over relevant exponential, ratio, and generalized regression estimators for intended domains.

## 1 Introduction

Missing information is an elegant issue in the sample survey. The missing information occurs when some individuals do not provide the desired information or fail to participate altogether. It can lead to the incomplete data and potential bias in the intend result. This missing information can be misses some units of the intended domains. If the units are missing in the data then it is termed to be the non-response. To mitigate non-response bias, survey agencies often employed various techniques. One approach is to be reported call backs it involves contracting the non-respondents multiple times to encourage their participation. This can be done through phone calls, reminder via email, or physical mail. By making repeated attempts ultimately to non-respondents, survey agencies aim to increase the response rate and reduce the potential bias caused by the non-response. There are many units are missing in the data it can be deal with the technique of the subsampling. In this technique, we are selected a sub sample by these non-respondent units.

It is not always possible that all observations on the plan variable are available. This may be because the information on the plan variable in the field studies is incomplete. Either the information was lost from the respondents due to some circumstance or the reviewer's questions were incorrect. The way to deal with non-response errors, at the beginning of the questionnaire, it is customary to include an introduction or some statements in the letters. The introduction clearly states the purpose of the survey, the motives for funding, and many more. The poll investigator humbly asks respondents to cooperate and provide help for relevant information. Some survey investigators give payments or gifts to respondents or raffle prizes among respondents to encourage participation in the survey. There are two types of missing information can occurs: i lost of the units ii lost of the items. The first is unit non-response, in which information about the unit is missing in the sample. The second is the item non-response, in which items were lost but those do not recollect by subsampling (for example over fertilization crop was destroy) from the non-respondent. It is mentioned that the difference between item and unit non-response. The plan variable can be obtained from the available respondent information. The remaining information can be collected by re-sampling either by email or by mail to the respondents. This will eliminate the remaining problem that still exists due to the non-response effects of the variable under study. If observations of the intended character have not been lost by chance, but perhaps prefixed. Therefore, it was estimated by the subsampling technique or, again, by answering questionnaires [1]. Various nonresponses [2] have explained using multistage sampling. Non-response has been estimated [[3], [4], [5], [6]] among others, who have worked in this area? Some references [[7], [8], [9]], and many others have applied the non-response method to the study variable. It can also be applied in the relevant areas that may be sub part of the population (subpopulation) for the intended character. These subpopulations are referred to as domains [10]. Based on the size, the article [11] has explained the types of domains: Major, minor, small or rare domains. It is very productive for real situations such as the income of the lower middle class, the income of the deprived areas, epidemiological issues, etc. Domains can be framed according to their practicality in the real scenario. Sometimes, domains need to be classified by the geographic area, e.g. districts, blocks, towns, and countries. Demographic characteristics such as gender, age, caste, socioeconomic characteristics (race-habits, income-race, income-employer, and caste-income), etc. represent different domains.

There are two methods of selection of the sample to the study domain: direct and indirect [12,13]. In the direct method, units are selected from the study area itself [14]. Domain estimation has been studied and explored using the supportive variable [15]. If supporting information is available regarding to the intended for small domain. The sufficient units present in the domain. It may be beneficial to the actual estimation of the small domain. If a large enough sample of intended variable falls in the domain, then the direct method of domain estimation provides efficient results. The accessible non-response in the study variable has been utilized by a known supportive variable [16]. Whenever, the small domains do not have sufficient units in the sample, the indirect method of domain estimation yields fruitful results. Also, the suitability of the variables is very helpful to obtained effective results.

In the present context, the problem becomes typical when non-response occurs in the context of small domain. The intensive effect of the non-response on the intensive variable can be reduced by supporting the re-sampling technique. The utility of the supporting variable is subject to constraints of the shorter distance of chi-squared applied during calibration. Calibration is an adjustment of the weights by the distance function [17,18]. Ref. [13] presented several techniques for estimating the total number of small domains. The work [4] has estimated domains and population totals in the presence of nonrespondent. Along these lines [19,20], have extended the utility of the supporting variable for estimating the domain total. Refs. [[21], [22], [23], [24]] have explained the generalized calibration without using the minimum chi-squared distance function. The minimum chi-square method is most convenient, suitable and most popular. They have also used the nonlinear distance function. Some of the situation, the variable under plan is present with unit non-response in the data, as mentioned earlier. This issue is introduced in this scenario, when the plan variable may be positively skewed. The supportive variable is of great importance, it should be considered for accessing the performance of the estimator. It has also been explained both the linear and nonlinear distance functions. The very interesting facet of domain estimation is the model-based approach. Recently, article [25] obtained an estimate of the domain sum using the indirect method using model-based approach. Now the opportunity is to consider the nature of the plan variable and supportive variable when we estimate domain total using appropriate model in the presence of unit non-respondent.

In the present study, we considered two conditions, accessible with and without presence of total of supporting variable of each small domain. The present study is the bud of the roots [[26], [27], [28]] in the above two conditions. We have given simulation study for the superiority of the power based models in both the situations.

The goals of the present work are describing for researchers. Why we develop third case? It illustrates simulation to check the performance of estimators empirically and an attempt to make work accessible in the holistic sense. Section 2 describes frame of the domains and their notations. Section 3 presents three cases of the proposed estimator if total of all domains of supportive variable response and non respondent are access. Section 4 discusses simulation using Newton Raphson approximation of the situation i. In Section 5, we discuss situation ii, in which we employ two phase sampling method. Estimate domain total under the remark 1 and remark 2. Section 6 describes a simulation study of the second situation. Section 7 describes some recommendations of both situations. Section 8 describes the applications of the proposed investigation which is an effort to make work to broad audience.

## 2 Problem presentation and their notations

We have a finite population U(1,2,3,...,N), for the plan we investigate non-overlapping L class (domains) $U_{a}\left(a=1,2,3,\ldots L\right)$ with size $N_{a}$. We plan of ath domain total $Y_{a}$. The population total and ath domain total of the auxiliary variable x are known. In the indirect method, a sample of size n is selected randomly by the population (size N). We categorized the respondent units and non-respondent units having sizes n_1_ and n_2_ respectively. We take a sub-sample from the non-respondent units n_2_ of size n_2r_ (n_2r_ = n_2_/k, k > 1). Hansen and Hurwitz [8] have been used a technique for estimation of the non-respondent. The work [2] has been explained the idea of the selection of sub-sample from the non-respondent. Now, we assume that unit $n^{*}$ consists of the respondent n_1_ unit of the sample s_1_ and sub-sample of the n_2r_ non-respondent of sample s_2r_ which is given in Equation (1). Where sample s_2r_ is sub-sample comes from the non-respondent sample s_2_.

(1) $$n^{*}=n_{1}+n_{2r}$$

Both y and x are given in Equation (2) and Equation (3)

(2) $$\sum_{i=1}^{n^{*}}d_{i}^{*}y_{i}^{*}=\sum_{i=1}^{n_{1}}d_{1i}y_{1i}+\sum_{i=1}^{n_{2r}}d_{1i}d_{2ri}y_{2ri}$$

And the corresponding unit of the respondent and non-respondent are given as

(3) $$\sum_{i=1}^{n^{*}}d_{i}^{*}x_{i}^{*}=\sum_{i=1}^{n_{1}}d_{1i}x_{1i}+\sum_{i=1}^{n_{2r}}d_{1i}d_{2ri}x_{2ri}$$

where, $\left(y_{i1},y_{2ri}\right)$ and $\left(x_{i1},x_{2ri}\right)$ denote the number of $n_{1}$ response and $n_{2r}$ number of sub-sample of non-responses of intended and helping variables y, and x, respectively.

The eminent values of y and x of the population and sample are as:

$N_{1}$ = Number of respondent in the population.
*N*_2_ = Number of non-respondent in the population.

Such that,

$$N_{1}+N_{2}=N$$

Y: Population total of the characteristic y based on the observations *N.*
X: Population total of the characteristic x based on the observations *N.*
$Y_{a}$: Total of intend variable ‘y’ of *a^th^* domain in the population based on $N_{a}$ observations.
$X_{a}$: Total of helping variable ‘x’ of *a^th^* domain in the population based on $N_{a}$ observations.
$y_{i}^{*}$: Sample value of plan variable y based on n* observations.
$x_{i}^{*}$: Sample value of supporting variable x based on n* observations.
*n*_*1*_ = Respondent units in $s_{1}$ sample.
$y_{i1}$: Sample of plan variable y having *n*_1_ respondent unit of $s_{1}$ sample.
$x_{i1}$: Sample of supporting variable x having *n*_1_ respondent unit of $s_{1}$ sample.
*n*_*2*_ = Non-respondent units in $s_{2}$ sample.
$y_{2r}$: Sub-sample observation of y from *n*_*2r*_ non-respondent unit of $s_{2r}$ sample.
$x_{2r}$: Sub-sample observation of x from *n*_*2r*_ non-respondent unit of $s_{2r}$ sample.

## 3 Methodology

The population U is classified into “a” non-overlapping different domains Ua of size N_a_ (a = 1, 2, 3, …, L). The supporting variable x is available for population and also for domains. A random sample of size n is selected from the population. The objective is to estimates the domain total of the plan variable $Y_{a}$. In a sample of size n, and $n_{a}$ units came from the domain Ua, where $n_{a}$ may be very small which can not give a reliable direct estimate of the domain total. So, it is essential to take an advantage of the surrounding units for estimation of the domain total. The main issue is to prepare an efficient estimate for plan of the domain total. It addition, aggregated information of x of all the domains are available. In the selected sample there are n_1_ respondent and sub-sample units $n_{2r}$ is equal to $n^{*}$. The plan of ath domain is based on the ith sample with probability $\pi_{i}^{*}=P_{r}\left\{i\in s\right\}\geq 0$ which consist of the respondent and non-respondent units with probability

$$\pi_{i}^{*}=\left\{ \begin{array}{c} \pi_{1i}\;i\in s_{1}\\ \pi_{1i}\pi_{2i}^{*}\;i\in s_{2} \end{array}\right.$$

where $\pi_{1i}$ shows ith units probability of respondent sample $s_{1}$ with size n_1_; and $\pi_{2i}$ shows i^th^ units probability of non respondent sample $s_{2}$. Actually, it comes from sub-sample of the non-respondent sample $s_{2r}$ with size n_2r_. The value of ${\pi^{\prime }}_{2i}$ is simply written by $\pi_{2i}^{*}=\pi_{2ri}/\pi_{2i}$. The article [28] has been discussed the calibration estimators using the auxiliary variable in two ways. However, the interest of the domain total has developed based on the geographic regions. The convenient units are the prime matter for the estimation purpose. The contribution of the indirect estimator with a single auxiliary variable has been applied on the ratio and generalized estimators, etc. The weights play a crucial role which utilizes the supportive variable in calibration approach. If weight will provide poor estimate then the estimator will be provide poor estimate. Hence, we should care of it. If weight will provide poor estimate then the estimator will be provide poor estimate. Hence, we should care of it. This is starting with the initial weight di*=1πi* that involves both weights respondent weight and nonrespondent weight, and these are denoted by d1i=1π1i and d′2i=1π2i/π1i , respectively. The sole work was developed by Ref. [29]. With help of this idea, we develop indirect estimator of ath domain total which is given by Equation (4)

(4) $$T_{P,a}^{*}=\sum_{i=1}^{n^{*}}t_{i}^{*}y_{i}^{*}=\sum_{i=1}^{n_{1}}t_{1i}y_{1i}+\sum_{i=1}^{n_{2r}}t_{2i}y_{2ri}\text{,}$$

where $t_{1i}$ and $t_{2i}$ are the respondent and non-respondent weights respectively. The auxiliary variable units corresponding to the plan variable of ath domain total should be known from the previous records. The supporting equation of the responseand non-response is given in Equation (5) and Equation (6)

(5) $$\sum_{i=1}^{n_{1}}t_{1i}x_{1i}=X_{1a}$$

and

(6) $$\sum_{i=1}^{n_{2r}}t_{2i}x_{2ri}=X_{2a}\text{.}$$

These are written by using a minimum chi-square distance in the following Equation (7)

(7) $$\frac{{\left(t_{1i}-d_{1i}\right)}^{2}}{2t_{1i}d_{1i}q_{1i}}+\frac{{\left(t_{2i}-d_{1i}{d^{\prime }}_{2i}\right)}^{2}}{2d_{1i}{d^{\prime }}_{2i}q_{2i}}\text{.}$$

The Lagrange's function can be written as in Equation (8)

(8) $$L\left(t_{1i},t_{2i},l_{1},l_{2}\right)=\sum_{i=1}^{n_{1}}\frac{{\left(t_{1i}-d_{1i}\right)}^{2}}{2d_{1i}q_{1i}}-l_{1}\left(X_{1a}-\sum_{i=1}^{n_{1}}t_{1i}x_{1i}\right)+\sum_{i=1}^{n_{2r}}\frac{{\left(t_{2i}-d_{1i}{d^{\prime }}_{2i}\right)}^{2}}{2d_{1i}{d^{\prime }}_{2i}q_{1i}}-l_{2}\left(X_{2a}-\sum_{i=1}^{n_{2r}}t_{2i}x_{2ri}\right)$$

where $l_{1}$ and $l_{2}$ are the respondent and non-respondent Lagrange's multipliers. The interaction is in getting the value of new weights $t_{1i}$, $t_{2i}$. The partially differentiate Equation (8) w.r.to two weights $t_{1i}$, $t_{2i}$ and equate to zero that is $\frac{\partial L\left(t_{1i},t_{2i},l_{1},l_{2}\right)}{\partial L\left(t_{1i}\right)}=0$ and $\frac{\partial L\left(t_{1i},t_{2i},l_{1},l_{2}\right)}{\partial L\left(t_{2i}\right)}=0$. After simplifying these two equations, we obtain respondent weight is given in Equation (9)

(9) $$t_{1i}=d_{1i}\left(1+l_{1}q_{1i}x_{1i}\right)$$

and non-respondent weight represent in Equation (10)

(10) $$t_{2i}=d_{1i}{d^{\prime }}_{2i}\left(1+l_{2}q_{2i}x_{2ri}\right)\text{.}$$

Substituting the values of $t_{1i}$ and $t_{2i}$ are in Equation (5) and Equation (6), respectively, we obtain the values of $l_{1}$ and $l_{2}$ which are mentioned in the following Equation (11) and Equation (12)

(11) $$l_{1}=\frac{\left(X_{1a}-\sum_{i=1}^{n_{1}}d_{1i}x_{1i}\right)}{\sum_{i=1}^{n_{1}}d_{1i}q_{1i}x_{1i}^{2}}$$

and

(12) $$l_{2}=\frac{\left(X_{2a}-\sum_{i=1}^{n_{2r}}d_{1i}{d^{\prime }}_{2i}x_{2ri}\right)}{\sum_{i=1}^{n_{2r}}d_{1i}{d^{\prime }}_{2i}q_{2i}x_{2ri}^{2}}\text{.}$$

The values of $l_{1}$ and $l_{2}$ are substituted in Equation (5), the response and non-respondent new weights are given in Equation (13) and Equation (14)

(13) $$t_{1i}=d_{1i}\left(1+\frac{\left(X_{1a}-\sum_{i=1}^{n_{1}}d_{1i}x_{1i}\right)}{\sum_{i=1}^{n_{1}}d_{1i}q_{1i}x_{1i}^{2}}q_{1i}x_{1i}\right)$$

and

(14) $$t_{2i}=d_{1i}{d^{\prime }}_{2i}\left(1+\frac{\left(X_{2a}-\sum_{i=1}^{n_{2r}}d_{1i}{d^{\prime }}_{2i}x_{2ri}\right)}{\sum_{i=1}^{n_{2r}}d_{1i}{d^{\prime }}_{2i}q_{2i}x_{2ri}^{2}}q_{2i}x_{2ri}\right)\text{.}$$

These new weights $t_{1i}$ and $t_{2i}$ are leaned in Equation (4), the developed estimator can be given by Equation (15)

(15) $$T_{P,a}^{*}=\sum_{i=1}^{n_{1}}d_{1i}y_{1i}+\sum_{i=1}^{n_{2r}}d_{1i}d_{2i}y_{2i}+\left(\frac{\sum_{i=1}^{n_{1}}d_{1i}q_{1i}x_{1i}y_{1i}}{\sum_{i=1}^{n_{1}}d_{1i}q_{1i}x_{2i}^{2}}\right)\left(X_{1a}-\sum_{i=1}^{n_{1}}d_{1i}x_{1i}\right)+\left(\frac{\sum_{i=1}^{n_{2r}}d_{1i}{d^{\prime }}_{2i}q_{2i}x_{2ri}y_{2ri}}{\sum_{i=1}^{n_{2r}}d_{1i}{d^{\prime }}_{2i}q_{2i}x_{2ri}^{2}}\right)\left(X_{2a}-\sum_{i=1}^{n_{2r}}d_{1i}{d^{\prime }}_{2i}x_{2ri}\right)\text{.}$$

**Case I:** If q1i=1x1i and q2i=1x2ri are included in Equation (15) then this equation will be ratio type estimator and is as in Equation (16)

(16) $$T_{Ratio,a}=\frac{\sum_{i=1}^{n_{1}}d_{1i}y_{1i}}{\sum_{i=1}^{n_{1}}d_{1i}x_{1i}}X_{1a}+\left(\frac{\sum_{i=1}^{n_{2r}}d_{1i}{d^{\prime }}_{2i}y_{2ri}}{\sum_{i=1}^{n_{2r}}d_{1i}{d^{\prime }}_{2i}x_{2ri}}\right)X_{2a}\text{.}$$

**Case II:** If $q_{1i}=1$ and $q_{2i}=1$ are put down in Equation (15), then the develop estimator will be converted to the regression type estimator like as Equation (17)

(17) $$T_{GREG,a}^{*}=\sum_{i=1}^{n_{1}}d_{1i}y_{1i}+\sum_{i=1}^{n_{2r}}d_{1i}d_{2i}^{\prime }y_{2i}+\frac{\sum_{i=1}^{n_{1}}d_{1i}x_{1i}y_{1i}}{\sum_{i=1}^{n_{1}}d_{1i}x_{1i}^{2}}\left(X_{1a}-\sum_{i=1}^{n_{1}}d_{1i}x_{1i}\right)+\frac{\sum_{i=1}^{n_{2r}}d_{1i}{d^{\prime }}_{2i}x_{2ri}y_{2ri}}{\sum_{i=1}^{n_{2r}}d_{1i}{d^{\prime }}_{2i}x_{2ri}^{2}}\left(X_{2a}-\sum_{i=1}^{n_{2r}}d_{1i}{d^{\prime }}_{2i}x_{2ri}\right)\text{.}$$

The new form of the regression estimatorcan be written in Equation (18) as

(18) $$T_{GREG,a}^{*}=\sum_{i=1}^{n^{*}}d_{i}^{*}y_{i}^{*}+{\hat{\alpha }}_{1}\left(X_{1a}-\sum_{i=1}^{n_{1}}d_{1i}x_{1i}\right)+{\hat{\alpha }}_{2}\left(X_{2a}-\sum_{i=1}^{n_{2r}}d_{1i}{d^{\prime }}_{2i}x_{2ri}\right)$$

where ${\hat{\alpha }}_{1}=\frac{\sum_{i=1}^{n_{1}}d_{1i}x_{1i}y_{1i}}{\sum_{i=1}^{n_{1}}d_{1i}x_{2i}^{2}}$ and ${\hat{\alpha }}_{2}=\frac{\sum_{i=1}^{n_{2r}}d_{1i}{d^{\prime }}_{2i}x_{2ri}y_{2ri}}{\sum_{i=1}^{n_{2r}}d_{1i}{d^{\prime }}_{2i}x_{2ri}^{2}}$.

The expectation of the GREG is given as Equation (19)

(19) $$E\left(T_{GREG,a}^{*}\right)=E\left(Y+{\hat{\alpha }}_{1}\left(X_{1a}-X_{1}\right)+{\hat{\alpha }}_{2}\left(X_{2a}-X_{2}\right)\right)\neq Y_{a}\text{.}$$

where ${\hat{X}}_{1}=\sum_{i=1}^{n_{1}}d_{1i}x_{1i}$ and ${\hat{X}}_{2}=\sum_{i=1}^{n_{2r}}d_{1i}{d^{\prime }}_{2i}x_{2ri}$.

Non respondent sample is not biased estimator of the non-respondent population $X_{2}$. Respondent sample is not biased estimator of the respondent population $X_{1}$. Since, sample is not biased estimator of the population total. The regression estimator is to be in Equation (19)

$$E\left(T_{GREG,a}^{*}\right)\neq Y_{a}\text{.}$$

The regression estimator $T_{GREG,a}^{*}$ is a biased estimator of the domain total.

In the same way, we can obtain ratio estimator isbiased estimator for the domain total.

**Case III:** If we put $q_{1i}\neq 1$ and $q_{2i}\neq 1$ in Equations (9), (10). Here, we can take the idea of function used by Refs. [30,31]. Equation (9) can be written in Equation (20) for respondent weights

(20) $$v_{1i}=d_{1i}\;\exp\left(l_{1}x_{1i}\right)\text{,}$$

where, exponential explanation given in Equation (21) and in Equation (22)

(21) $$\exp\left(l_{1}x_{1i}\right)=1+\frac{\left(l_{1}x_{1i}\right)}{1!}+\frac{{\left(l_{1}x_{1i}\right)}^{2}}{2!}+\frac{{\left(l_{1}x_{1i}\right)}^{3}}{3!}+...=1+l_{1}x_{1i}\left(1+\frac{\left(l_{1}x_{1i}\right)}{2!}+\frac{{\left(l_{1}x_{1i}\right)}^{2}}{3!}+...\right)=1+l_{1}x_{1i}q_{1i}$$

where,

(22) $$q_{1i}=1+\frac{\left(l_{1}x_{1i}\right)}{2!}+\frac{{\left(l_{1}x_{1i}\right)}^{2}}{3!}+...$$

Equation (10) denotes respondent weight same to Equation (23) that can be write in the new function form

(23) $$v_{2i}=d_{1i}{d^{\prime }}_{2i}\;\exp\left(l_{2}x_{2ri}\right)$$

where, the derivation of exponential is given in Equation (24) and their constant in Equation (25)

(24) $$\exp\left(l_{2}x_{2ri}\right)=1+\frac{\left(l_{2}x_{2ri}\right)}{1!}+\frac{{\left(l_{2}x_{2ri}\right)}^{2}}{2!}+\frac{{\left(l_{2}x_{2ri}\right)}^{3}}{3!}+...=1+l_{2}x_{2ri}\left(1+\frac{\left(l_{2}x_{2ri}\right)}{2!}+\frac{{\left(l_{2}x_{2ri}\right)}^{2}}{3!}+...\right)=1+l_{2}x_{2ri}q_{2i}$$

where,

(25) $$q_{2i}=1+\frac{\left(l_{2}x_{2ri}\right)}{2!}+\frac{{\left(l_{2}x_{2ri}\right)}^{2}}{3!}+...$$

The values of $l_{1}$ and $l_{2}$ can be estimated with approximation technique of Newton-Raphson method. Other method can be used like method of scoring [32]. If estimated value of the new weights $v_{1i}$ and $v_{2i}$ are substituted in Equation (4), then the exponential estimator in the following Equation (26)

(26) $${T_{NG}^{*}}_{,a}=\sum_{i=1}^{n_{1}}d_{1i}y_{1i}\;\exp\left(l_{1}x_{1i}\right)+\sum_{i=1}^{n_{2r}}d_{1i}{d^{\prime }}_{2i}y_{2ri}\;\exp\left(l_{2}x_{2ri}\right)\text{.}$$

There are many models which keep similar in the nature of exponential. They have positively skew like power type model. In the real context, such situation can be looked for income of households, or average income of the families in a specific region. Therefore, we are presenting a power type model. Equation (27) shows power weight function of respondent's weights is

(27) $$u_{1i}=d_{1i}\eta_{1}^{x_{1i}}\text{.}$$

It can be expressed in the following form written in Equation (28), Equation (29) and Equation (30)

(28) $${\left(\eta_{1}\right)}^{x_{1i}}=1+\eta_{1}x_{1i}\left\{-\left(\frac{1}{\eta_{1}}-1\right)+\frac{\left(x_{1i}-1\right)\left(1-\eta_{1}\right)\left(\frac{1}{\eta_{1}}-1\right)}{2!}-\frac{\left(x_{1i}-1\right)\left(x_{1i}-2\right){\left(1-\eta_{1}\right)}^{2}\left(\frac{1}{\eta_{1}}-1\right)}{3!}+...\right\}$$

where, the value of $q_{1i}$.

(29) $$q_{1i}=\left\{-\left(\frac{1}{\eta_{1}}-1\right)+\frac{\left(x_{1i}-1\right)\left(1-\eta_{1}\right)\left(\frac{1}{\eta_{1}}-1\right)}{2!}-\frac{\left(x_{1i}-1\right)\left(x_{1i}-2\right){\left(1-\eta_{1}\right)}^{2}\left(\frac{1}{\eta_{1}}-1\right)}{3!}+...\right\}\text{.}$$

Thus,

(30) $$u_{1i}=d_{1i}\left(1+\eta_{1}q_{1i}x_{1i}\right)\text{.}$$

Similarly, the non-respondent weight function is in Equation (31)

(31) $$u_{2i}=d_{1i}{d^{\prime }}_{2i}\eta_{2}^{x_{2ri}}\text{.}$$

It can be explained in Equation (32)

(32) $${\left(\eta_{2}\right)}^{x_{2ri}}=1+\eta_{2}x_{2ri}\left\{-\left(\frac{1}{\eta_{2}}-1\right)+\frac{\left(x_{2ri}-1\right)\left(1-\eta_{2}\right)\left(\frac{1}{\eta_{2}}-1\right)}{2!}-\frac{\left(x_{2ri}-1\right)\left(x_{2ri}-2\right){\left(1-\eta_{2}\right)}^{2}\left(\frac{1}{\eta_{2}}-1\right)}{3!}+...\right\}$$

where, the value of chosen constant is

(33) $$q_{2i}=\left\{-\left(\frac{1}{\eta_{2}}-1\right)+\frac{\left(x_{2ri}-1\right)\left(1-\eta_{2}\right)\left(\frac{1}{\eta_{2}}-1\right)}{2!}-\frac{\left(x_{2ri}-1\right)\left(x_{2ri}-2\right){\left(1-\eta_{2}\right)}^{2}\left(\frac{1}{\eta_{2}}-1\right)}{3!}+...\right\}\text{.}$$

After simplifying Equation (32) and Equation (33), it can be written by Equation (34)

(34) $$u_{2i}=d_{1i}{d^{\prime }}_{2i}\left(1+\eta_{2}q_{2i}x_{2ri}\right)\text{.}$$

It is put down in Equation (4), Equation (35) will be power function type estimator

(35) $${T_{Power}^{*}}_{,a}=\sum_{i=1}^{n_{1}}d_{1i}y_{1i}\eta_{1}^{x_{1i}}+\sum_{i=1}^{n_{2r}}d_{1i}{d^{\prime }}_{2i}y_{2ri}\eta_{2}^{x_{2ri}}\text{.}$$

The obtain value of the bias and mean square error of power function. The expectation of the power-based estimator of the domain total is

$$E\left({T_{Power}^{*}}_{,a}\right)\neq Y_{a}\text{.}$$

It means develop estimator is biased. It is asymptotically shows that model-based estimate converges to the estimate of design-based. It has been obtained that ratio and regression estimators are biased. Hence, the developed estimate is also biased of total of domain. The model and design-based estimators are biased; hence their performance can be shown through empirically. It is estimating through simulation study in the terms of absolute relative bias and simulated relative standard error.

## 4 Simulation

The simulation provides major concern of showing the impact of estimators using real data set. For simulation, a population is sufficient to explain the work. The nature should hold for the study purpose. It took a real data from the Swedish municipality [33]. There are two important reasons of taking six domains one is the different unit access in the domains. Second is the nature of these domains are positively skewed in nature. The areas 1, 2, 3, 4, 5 and 6 with sizes of 48, 32, 38, 41, 15 and 29, respectively, were considered for the study. It has seen that the nature of the data is positively skewed. Moreover, the non-response rate is assumed to be approximately the same in the domain and in the total population. Here, we selected a sample size 70 of the population unit 203. Approximately 30 percentage of the non-response units, i.e. 21, and rest are response units, of which 49 are left. It was verified that the average the response and non-response units is 49 and 21, respectively. It has used two different non-response units n_2r_ = 12 and 15. This process is repeated up to 15,000 times, since these are subsets of the total possible sample combination without replacement of 70 out of 203. In the first case small 30, 40 and 50 sizes sample do not provide better result to 70 and 90 sizes. Later, it is considered another sample which has size 90, to show the effect of increase sample size on the estimated value. In this case, the response and non-response unitsare 63 and 27, respectively. The non-response is the same as the previous sample. The same process can be employed using well known distribution which has similar nature like real data. But, we have real data set, so can do this data set.

It was employed from study and auxiliary variables. Indirect method-based discussed estimators Nguyen $\left(T_{NG,a}^{*}\right)$, power $\left(T_{Power,a}^{*}\right)$, ratio $\left(T_{Ratio,a}^{*}\right)$ and generalized regression $\left(T_{GREG,a}^{*}\right)$ of intend domain.

Both variables y and x are given as follows in Table 1:

y: Number of municipal employees in 1984.
x: Real estate values according to 1984 assessment (in millions of Kronor).

**Table 1:** The value of respondent and non-respondent of the y and x and their sizes.

Parameter	Domains					
$N_{a}$	48	32	38	41	15	29
$N_{1a}$	34	22	27	29	10	20
$N_{2a}$	14	10	11	12	5	9
$Y_{a}$	142,613	79,960	110,790	89,188	54,727	65,804
$Y_{1a}$	101034.6	54976.49	78626.42	63072.35	36483.44	45356.59
$Y_{2a}$	41578.45	24983.51	32163.58	26115.65	18243.56	20447.41
$X_{a}$	79,618	42,145	73,633	45,090	23,008	30,631
$X_{1a}$	56477.09	28974.45	52447.71	31919.81	15322.23	21187.13
$X_{2a}$	23140.91	13170.55	21185.29	13170.19	7685.77	9443.86

Equation (36) and Equation (37) show absolute relative bias (ARB) and simulate relative standard error (SRSE) of the conventional estimators are as follows

(36) $$ARB\left(T_{L,a}^{*}\right)=\frac{\left|\sum_{s=1}^{15000}T_{L,a}^{*s}-Y_{a}\right|}{Y_{a}}*100$$

(37) $$SRSE\left(T_{L,a}^{*s}\right)=\frac{\sqrt{SMSE\left(T_{L,a}^{*s}\right)}}{Y_{a}}$$

where, simulated mean squared error is given in Equation (38)

(38) $$SMSE\left(T_{L,a}^{*s}\right)=\frac{1}{15000}\sum_{s=1}^{15000}{\left(T_{L,a}^{*s}-Y_{a}\right)}^{2}$$

$T_{L,a}^{*s}$: sth sample of the estimators $T_{GREG,a}^{*}$, $T_{Ratio,a}^{*}$, $T_{NG,a}^{*}$ and $T_{Power,a}^{*}$ for *a*th domain.

From Table 2, the SRSE value of indirect power, Nguyen, ratio and, GREG ranged (19.27, 42.51), (20.35, 44.26), (21.95, 49.87) and (70.78, 153.91) for the domains (1, 2, 3, 4, 5 and 6), respectively (when n2r = 12 and n = 70). The power based estimate is shortest ranged from all the estimators. It is also found that all the estimators provide a poor estimate for domain 5. This is due to the effects of non-response estimate and low unit availability in the planed domain. Whenever, for domain 2 the estimate is slightly better as compared to the domain 5. The performance of power-based estimates is the lowest among all the estimators in terms of the absolute relative bias. The significant point is analysed that the power based estimate is more precise estimates when increase the sub-sample from the nonresponse sample. It gets different results as the sample of size 90 than for the sample of size 70. The value of ARB is relatively different for different nonresponse values of ARB when the sample size is 70 which due to variationin the response and sub-sample of non-response units. Due to bias the absolute relative is wide but simulates relative standard error is decreasing. We have taken care of sensitiveness of domain and population information, because estimator can not away from the main theme of the paper. The resultis verifying [2] estimator. Finally, it is concluded that the power estimates perform better than the other estimators for both sample sizes low non-response sub-sample for all the estimators. For sample of size 90, we obtained the results are different than the sample of size 70.

**Table 2:** The absolute relative bias and simulated relative standard error using minimum chi-square distance with 30% non-response of regression, ratio, Nguyen and power estimator $T_{GREG,a}^{*}$, $T_{Ratio,a}^{*}$, $T_{NG,a}^{*}$ and $T_{Power,a}^{*}$ for the domains 1, 2, 3, 4, 5 and 6.

Sample Size	Estimators	Domains					
n		n_2r_ = 12					
		1	2	3	4	5	6
70	$T_{GREG,a}^{*}$	74.21 (58.29)	128.97 (100.58)	71.37 (31.96)	81.34 (35.27)	150.34 (102.21)	101.25 (50.63)
	$T_{Ratio,a}^{*}$	28.23 (10.49)	27.23 (8.72)	47.35 (34.12)	21.87 (0.30)	48.25 (36.23)	22.04 (7.02)
	$T_{NG,a}^{*}$	26.03 (8.09)	25.47 (7.67)	45.12 (29.89)	19.57 (0.26)	45.69 (30.78)	20.97 (3.13)
	$T_{Power,a}^{*}$	23.78 (7.68)	23.37 (6.52)	41.04 (27.76)	18.34 (0.22)	41.95 (28.96)	19.29 (2.91)
	n_2r_ = 15						
	$T_{GREG,a}^{*}$	54.25 (25.68)	124.26 (95.89)	58.28 (18.35)	75.28 (22.07)	136.56 (42.05)	96.42 (40.15)
	$T_{Ratio,a}^{*}$	26.22 (10.09)	26.69 (8.34)	44.82 (36.23)	26.27 (5.12)	47.21 (32.13)	21.23 (5.61)
	$T_{NG,a}^{*}$	24.79 (8.02)	24.89 (7.87)	40.13 (32.57)	25.13 (3.56)	42.75 (29.79)	20.21 (4.21)
	$T_{Power,a}^{*}$	23.12 (7.42)	22.57 (5.98)	38.17 (29.41)	23.26 (3.46)	39.27 (27.31)	19.04 (2.84)
	n_2r_ = 14						
90	$T_{GREG,a}^{*}$	55.25 (21.21)	86.44 (34.73)	70.74 (33.36)	78.95 (32.16)	86.90 (61.80)	103.70 (51.79)
	$T_{Ratio,a}^{*}$	31.30 (12.70)	26.16 (5.73)	44.98 (32.63)	24.05 (1.06)	45.85 (32.68)	22.45 (7.51)
	$T_{NG,a}^{*}$	28.35 (11.59)	24.34 (4.14)	41.67 (30.23)	22.58 (0.89)	40.23 (28.89)	20.11 (6.79)
	$T_{Power,a}^{*}$	27.45 (10.78)	21.23 (3.67)	37.34 (27.68)	20.23 (0.77)	37.49 (25.34)	18.45 (5.78)
	n_2r_ = 19						
	$T_{GREG,a}^{*}$	47.70 (15.64)	73.58 (20.77)	62.79 (23.60)	71.54 (25.65)	60.43 (46.91)	96.93 (48.41)
	$T_{Ratio,a}^{*}$	25.97 (11.97)	23.12 (6.92)	42.35 (32.07)	21.61 (2.48)	43.27 (31.63)	21.16 (8.17)
	$T_{NG,a}^{*}$	24.23 (10.34)	21.67 (6.12)	38.23 (28.68)	20.45 (2.25)	40.27 (28.34)	20.04 (7.76)
	$T_{Power,a}^{*}$	22.67 (8.67)	19.02 (5.67)	34.67 (24.67)	18.98 (2.10)	37.67 (25.67)	19.14 (7.31)

(): shows absolute relative bias for conventional indirect estimators.

## 5 Two phase calibration approach

In practice, the availability of supporting information is crucial and significant impact on the performance of the estimators. When supportive variable is present, that helps in the improvement of the accuracy of the domain estimation. However, the sum of the supporting variable is not known. This leads to problems in estimating the domain parameter. The calibrated estimators used the calibration process if supporting variable total is not known. Hence, create trouble during the estimates of the parameter of the calibrated estimators under calibration. In this case, two phase sample may provide a better estimate of the parameter was developed by Ref. [34]. The first phase sample is used to collect at the domain total of helping variable, however second phase estimate is primary interest of domain total which was used for domain total of the population level. This approach allows for estimation of the domain total by utilizing the information from both phases of the sample. By introducing the information is in the study variable. The two phase sample approach can help to mitigate the problems arising from the unknown sum of the supportive variable. It enables more accurate estimation of the parameters of the calibrated estimates. Non-response in the domain is another major concerned of the accessible domain total. The works [35,36] have explained the non-response and two-phase method with a calibration approach. Due to loss of information in the different areas, the above idea can be used. The information of first phase plays a crucial role for the second phase. This is also evident in the present work. The variations in the supporting information for the population are also leading to variations in the helping information. However, the question arises as to how much the first-phase information affects the second-phase sample and the calibration approach? These works [10,28,37] have presented the two phase calibration approach. Recently, article [32] extended the indirect two phase calibration approach to include a model weighting function for range estimation.

Indeed the total value of helping variable is not easily accessible or in same circumstances times unit-level data are not available. Therefore, it can use a two phase sampling technique.To gets an estimate of the total of the supporting variables for the domain. It estimates by using the calibration weights of both phases sample. Thus, let us take a population with N countable units Ω. The pre-define domain have $N_{a}$ units of $\Omega_{a}$ which make a population of Ω in total. A random sample $s_{1}^{\prime }:\{s_{1}^{\prime }\subseteq \Omega \}$ is chosen of the first phase with known sampling design. The response probability of first phase sample $s_{11}^{\prime }:\left\{s_{11}^{\prime }\subseteq s_{1}^{\prime }\right\}$ of size $n_{11}^{\prime }$ is $\pi_{11i}^{\prime }=P_{r}\{i\in s_{11}^{\prime }\}$. The probability of the sub-sample of non-response at the first phase sample $s_{12r}^{\prime }:\left\{s_{12r}^{\prime }\subseteq s_{11}^{\prime }\right\}$ of size $n_{12r}^{\prime }$ is $\pi_{12ri}^{\prime }=P_{r}\{i\in s_{12}^{\prime }\}$. The second phase sample is sub-sample selected from the first phase sample $s_{11}^{\prime \prime }:\{s_{11}^{\prime \prime }\subseteq s_{11}^{\prime }\subseteq \Omega \}$ has size $n_{11}^{\prime \prime }$. The probability of the response at second phase sample $s_{11}^{\prime \prime }:\left\{s_{11}^{\prime \prime }\subseteq s_{1}^{\prime }\right\}$ is $\pi_{11i}^{\prime \prime }=P_{r}\{i\in s_{11}^{\prime \prime }\}$. The probability of sub-sample of non-response of second phase sample $s_{12r}^{\prime \prime }:\left\{s_{12r}^{\prime \prime }\subseteq s_{12}^{\prime \prime }\right\}$ of size $n_{12r}^{\prime \prime }$ is $\pi_{12ri}^{\prime \prime }=P_{r}\{i\in s_{12}^{\prime \prime }\}$. The probability of ith unit of ath domain of the sample has probability $\pi_{i}^{*}=P_{r}\left\{i\in s\right\}\geq 0$ which consist both response and non-response units with probabilities

$${\pi^{\prime }}_{1i}^{*}=\left\{ \begin{array}{c} {\pi^{\prime }}_{11i}\;i\in {s^{\prime }}_{11}\\ {\pi^{\prime }}_{11i}{\pi^{\prime }}_{12ri}\;i\in {s^{\prime }}_{12} \end{array}\right.$$

The response of first phase weights represented as d11i′=1π11i′ and non-response of first phase weights represented by d12ri′=1π12ri/π12i′′. A second phase weights of the response and sub-sample by the non-response at second phase weights are represented d11i″=1π11i″ and d12ri″=1π12ri/π12i′″ respectively. The eventual weight involved first and second weights. The eventual weightis the multiplication of both phases weights. The domain total value will be ${\hat{Y}}_{a}=\sum_{n_{2}}t_{2i}^{\prime *}t_{2i}^{\prime \prime }y_{i}^{\prime }$. At each phase response and non-response units are available in advance. The second phase units come from the first phase units. The classification of both phase weights has been classified of *i*th unit of helping variable as $x_{i}=\left(x_{1i}^{\prime },x_{2i}^{\prime }\right)$ in Table 3.

**Table 3:** The classification of auxiliary variable of *i*th units at both phases.

Sets	Accessible units		
	Total	Response	Non-response
Population	$x_{i};i\in s_{i}\;or\;\sum_{i=1}^{\Omega }x_{i}$	$x_{i}^{\prime };i\in s_{1}^{\prime }$	$x_{i}^{\prime \prime };i\in s_{2}^{\prime }$
First Phase Sample	$x_{i}^{\prime };i\in s_{1}^{\prime }$	$x_{11i}^{\prime };i\in s_{11}^{\prime }$	$x_{12ri}^{\prime };i\in s_{12}^{\prime }$
Second Phase Sample	$x_{i}^{\prime \prime };i\in s_{1}^{\prime \prime }$	$x_{11i}^{\prime \prime };i\in s_{11}^{\prime \prime }$	$x_{12ri}^{\prime \prime };i\in s_{12}^{\prime \prime }$

The simple classification of the both phases of the auxiliary variable with their justifications is given as:

Remark 1 In the two phase sampling, it decides on the helping variable according to the needs and easy availability. Then it decided the selection of *i*th unit of helping variable in the three forms (a) $x_{i}$ can be written as $x_{i}=\left(x_{i}^{\prime },x_{i}^{\prime \prime }\right)$; (b) $x_{i}=x_{i}^{\prime }$ and (c) $x_{i}=x_{i}^{\prime \prime }$. It starts with first phase sample. The last form was elaborated [26]. Without loss of generality, we assume that $x_{i}^{\prime \prime }$ is equivalent to $x_{i}^{\prime }$. The form of second phase variable is equal to $x_{i}=x_{i}^{\prime \prime }$, to convince the information in the vector form of first and second phase in the article [38].

Remark 2 The calibration weights of the vectors form lead $t_{i}^{\prime *}$ from $s_{2}^{\prime }$ sample is obtained from the initial phase sample $s_{1}^{\prime }$. The simplest form of initial phase and second phase equation are equal to $\sum_{i=1}^{n_{1}^{\prime \prime }}t_{i}^{\prime \prime }x_{i}^{\prime }=\sum_{i=1}^{n_{1}^{\prime }}t_{i}^{\prime }x_{i}^{\prime }$. Some variation can be happened between the both phases sample units. The initial phase sample units are reliable which is depending on the available units in the domains. If there are small units in the domain so, increase the first phase sample units for efficient results. We have estimated the domain total by $\sum_{i=1}^{n_{1}^{\prime \prime }}t_{i}^{\prime \prime }y_{i}^{\prime }$.

(i)First Phase Sampling

The new weights of response and non-response weights of the initial phase samples are $\left\{t_{11i}^{\prime };i\in s_{11}^{\prime }\right\}$ and $\left\{t_{2ri}^{\prime };i\in s_{12}^{\prime }\right\}$. For the initial phase sample of the population, the distance function likes to be Equation (39)

(39) $$D_{1}=\frac{1}{2}\sum_{i=1}^{n_{11}^{\prime }}\frac{{\left(t_{11i}^{\prime *}-t_{11i}^{\prime }\right)}^{2}}{t_{11i}^{\prime }{q^{\prime }}_{11i}}+\frac{1}{2}\sum_{i=1}^{n_{12r}^{\prime }}\frac{{\left(t_{12i}^{\prime *}-t_{11i}^{\prime }t_{12ri}^{\prime }\right)}^{2}}{t_{11i}^{\prime }t_{12ri}^{\prime }{q^{\prime }}_{12i}}$$

where, $t_{11i}^{\prime }$, $t_{12ri}^{\prime }$ are design weights and $t_{11i}^{\prime *}$ is updated weight.

The initial phase calibration equation is written by Equation (40)

(40) $$\frac{1}{2}\sum_{i=1}^{n_{11}^{\prime }}t_{11i}^{\prime *}x_{11i}^{\prime }+\frac{1}{2}\sum_{i=1}^{n_{12r}^{\prime }}t_{11i}^{\prime *}t_{12ri}^{\prime *}x_{12ri}^{\prime }=x_{11,a}^{\prime }$$

where, $x_{11,a}^{\prime }$ shows *a*th domain total of helping variable which is obtained from the sample of initial phase.

(ii) Double Phase Sampling

The initial phase weights $t_{1i}^{\prime *}$ is obtained by the utility of population level previous information in hand. It emphasises for sample of final phase of plan variable $\left\{y_{i}^{\prime };i\in s_{1}^{\prime \prime }\right\}$. It is introduced at the estimation as simply $\hat{Y}=\sum_{i=1}^{s_{1}^{\prime \prime }}t_{11i}^{*}t_{12i}^{\prime *}y_{i}^{\prime *}$ contains weights of $x_{2i}$. The sample $\left\{{q^{\prime }}_{12i};i\in s_{2}^{\prime }\right\}$ is used to estimate second (final) phase weights. Equation (41) is minimum chi square distance

(41) $$D_{2}=\frac{1}{2}\sum_{i=1}^{n_{11}^{\prime \prime }}\frac{{\left(t_{11i}^{\prime \prime *}-t_{11i}^{\prime *}d_{11i}^{\prime \prime }\right)}^{2}}{t_{11i}^{\prime *}{d^{\prime \prime }}_{11i}{q^{\prime }}_{12i}}+\frac{1}{2}\sum_{i=1}^{n_{12r}^{\prime \prime }}\frac{{\left(t_{12i}^{\prime \prime }-t_{12ri}^{\prime }{d_{11i}^{\prime \prime }d^{\prime \prime }}_{12ri}\right)}^{2}}{t_{12ri}^{\prime }{d_{11i}^{\prime \prime }d^{\prime \prime }}_{12ri}{q^{\prime }}_{12i}}\text{.}$$

This distance is used for assumption of helping variable like Equation (42)

(42) $$\sum_{i=1}^{n_{1}^{\prime \prime }}t_{i}^{\prime *}x_{i}^{\prime }=\sum_{i=1}^{n_{1}^{\prime }}t_{11i}^{\prime *}x_{i}^{\prime }\text{.}$$

It is further used the estimation of total of population $T_{P}=\sum_{i=1}^{n_{1}^{\prime \prime }}{\tilde{t}}_{i}^{\prime *}y_{i}^{\prime }$.

Now, we develop estimator of domain total using two phase in the presence of unit non-response. The developed estimator using two phase sample of ath domain total given by Equation (43)

(43) $$T_{P,a}^{*}=\sum_{i=1}^{n^{*}}t_{i}^{*}y_{i}^{*}=\sum_{i=1}^{n_{1}^{\prime \prime }}t_{11i}^{\prime \prime *}y_{11i}^{\prime }+\sum_{i=1}^{n_{2r}^{\prime \prime }}t_{12i}^{\prime \prime *}y_{12i}^{\prime }\text{,}$$

where $t_{11i}^{\prime \prime *}$ and $t_{12i}^{\prime \prime *}$ represent the response and non-response weights. The helping variable corresponding to a^th^ domain does not accessible easily. Then the response and non-response supporting Equation (44) and Equation (45) are given by

(44) $$\sum_{i=1}^{n_{11}^{\prime }}t_{11i}^{\prime *}x_{11i}^{\prime }=x_{1a}^{\prime }$$

and

(45) $$\sum_{i=1}^{n_{12r}^{\prime }}t_{12i}^{\prime *}x_{12i}^{\prime }=x_{2a}^{\prime }\text{.}$$

We are using minimum chi-square distance function under remark 1 as Equation (46)

(46) $$D_{1}\left(t_{11i}^{\prime *},{d^{\prime }}_{11i}\right)=\frac{1}{2}\sum_{i=1}^{n_{11}^{\prime }}\frac{{\left(t_{11i}^{\prime *}-d_{11i}^{\prime }\right)}^{2}}{d_{11i}^{\prime }{q^{\prime }}_{11i}}+\frac{1}{2}\sum_{i=1}^{n_{12r}^{\prime }}\frac{{\left(t_{12i}^{\prime *}-d_{11i}^{\prime }d_{12ri}^{\prime }\right)}^{2}}{d_{11i}^{\prime }d_{12ri}^{\prime }{q^{\prime }}_{12i}}$$

The calibration equation is the sum of the shortest distance and helping variable restriction at first phase of response and non-response units like Equation (47)

(47) $$L\left(t_{11i}^{\prime *},t_{12i}^{\prime *},\varsigma_{1},\varsigma_{2}\right)=\sum_{i=1}^{n_{11}^{\prime }}\frac{{\left(t_{11i}^{\prime *}-d_{11i}^{\prime }\right)}^{2}}{2d_{11i}^{\prime }{q^{\prime }}_{11i}}+\varsigma_{1}\left(x_{1a}^{\prime }-\sum_{i=1}^{n_{11}^{\prime }}t_{11i}^{\prime *}{x^{\prime }}_{11i}\right)+\sum_{i=1}^{n_{12r}^{\prime }}\frac{{\left(t_{12i}^{\prime *}-d_{11i}^{\prime }d_{12ri}^{\prime }\right)}^{2}}{2d_{11i}^{\prime }d_{12ri}^{\prime }{q^{\prime }}_{12i}}+\varsigma_{2}\left(x_{2a}^{\prime }-\sum_{i=1}^{n_{12r}^{\prime }}t_{12i}^{\prime *}x_{12ri}^{\prime *}\right)$$

where $\varsigma_{1}$ and $\varsigma_{2}$ are the response and non-response Lagrange's multipliers. We are interested in the value of new weights $t_{11i}^{\prime *}$ and $t_{12i}^{\prime *}$. Partially differentiating Equation (47) w.r.to two weights $t_{11i}^{\prime *}$, $t_{12i}^{*}$ and equating to $\frac{\partial L\left(t_{11i}^{\prime *},t_{12i}^{\prime *},\varsigma_{1},\varsigma_{2}\right)}{\partial L\left(t_{11i}^{\prime *}\right)}=0$ and $\frac{\partial L\left(t_{11i}^{\prime *},t_{12i}^{\prime *},\varsigma_{1},\varsigma_{2}\right)}{\partial L\left(t_{12i}^{\prime *}\right)}=0$. We write response weight in Equation (48)

(48) $$t_{11i}^{\prime *}=d_{11i}^{\prime *}\left(1+\varsigma_{1}{q^{\prime }}_{11i}{x^{\prime }}_{11i}\right)$$

and non-response weight in Equation (49)

(49) $$t_{12i}^{\prime *}=d_{11i}^{\prime }d_{12ri}^{\prime }\left(1+\varsigma_{2}{q^{\prime }}_{12i}{x^{\prime }}_{12ri}\right)\text{.}$$

It put down both values $t_{11i}^{\prime *}$ and $t_{12i}^{\prime *}$ in Equation (44) and Equation (45) respectively, we obtain the values of $\varsigma_{1}$ and $\varsigma_{2}$ as in Equation (50) and Equation (51)

(50) $$\varsigma_{1}=\frac{\left(x_{1a}^{\prime }-\sum_{i=1}^{{n^{\prime }}_{11}}d_{11i}^{\prime }{x^{\prime }}_{11i}\right)}{\sum_{i=1}^{{n^{\prime }}_{11}}d_{11i}^{\prime }{q^{\prime }}_{11i}x_{11i}^{\prime 2}}$$

and

(51) $$\varsigma_{2}=\frac{\left(x_{2a}^{\prime }-\sum_{i=1}^{{n^{\prime }}_{12r}}d_{11i}^{\prime }d_{12ri}^{\prime }{x^{\prime }}_{12ri}\right)}{\sum_{i=1}^{{n^{\prime }}_{12r}}d_{11i}^{\prime }d_{12ri}^{\prime }{q^{\prime }}_{12i}x_{12ri}^{\prime 2}}\text{.}$$

Now, the value of $\varsigma_{1}$ and $\varsigma_{2}$ are substituting in Equation (48) and Equation (49), we obtain response in Equation (52) and non-response weights in Equation (53)

(52) $$t_{11i}^{\prime *}=d_{11i}^{\prime }\left(1+\frac{\left(x_{1a}^{\prime }-\sum_{i=1}^{{n^{\prime }}_{11}}d_{11i}^{\prime }{x^{\prime }}_{11i}\right)}{\sum_{i=1}^{{n^{\prime }}_{11}}d_{11i}^{\prime }{q^{\prime }}_{11i}{x^{\prime }}_{11i}^{2}}{q^{\prime }}_{11i}{x^{\prime }}_{11i}\right)$$

and

(53) $$t_{12i}^{\prime *}=d_{11i}^{\prime }d_{12ri}^{\prime \prime }\left(1+\frac{\left(x_{2a}^{\prime }-\sum_{i=1}^{{n^{\prime }}_{12r}}d_{11i}^{\prime }d_{12ri}^{\prime }{x^{\prime }}_{12ri}\right)}{\sum_{i=1}^{{n^{\prime }}_{12r}}d_{11i}^{\prime }d_{12ri}^{\prime }{q^{\prime }}_{12i}x_{12ri}^{\prime 2}}{q^{\prime }}_{12i}{x^{\prime }}_{12ri}\right)\text{.}$$

The new weight ${t^{\prime }}_{11i}^{*}$ and $t_{12i}^{\prime *}$ are substituted in Equation (43), Equation (54) write the proposed estimator is

(54) $${T^{\prime }}_{P,a}^{*}=\sum_{i=1}^{n_{11}^{\prime }}d_{11i}^{\prime }y_{11i}^{\prime }+\sum_{i=1}^{n_{12r}^{\prime }}d_{12ri}^{\prime }y_{12ri}^{\prime }+\left(\frac{\sum_{i=1}^{n_{11}^{\prime }}d_{11i}^{\prime }q_{11i}^{\prime }x_{11i}^{\prime }y_{11i}^{\prime }}{\sum_{i=1}^{n_{11}^{\prime }}d_{11i}^{\prime }q_{11i}^{\prime }{x^{\prime }}_{12i}^{2}}\right)\left(x_{1a}^{\prime }-\sum_{i=1}^{n_{11}^{\prime }}d_{11i}^{\prime }x_{11i}^{\prime }\right)+\left(\frac{\sum_{i=1}^{{n^{\prime }}_{12r}}d_{11i}^{\prime }d_{12ri}^{\prime }{q^{\prime }}_{12i}{x^{\prime }}_{12ri}{y^{\prime }}_{12ri}}{\sum_{i=1}^{{n^{\prime }}_{12r}}d_{11i}^{\prime }d_{12ri}^{\prime }{q^{\prime }}_{12i}{x^{\prime }}_{12ri}^{2}}\right)\left(x_{2a}^{\prime }-\sum_{i=1}^{{n^{\prime }}_{12r}}d_{11i}^{\prime }d_{12ri}^{\prime }{x^{\prime }}_{12ri}\right)\text{.}$$

**Case I:** If q′11i=1x′11i and q′12i=1x′12ri are put down in Equation (54), then the proposed estimator become to be the ratio type estimator like Equation (55)

(55) $$T_{Ratio,a}^{\prime *}=\frac{\sum_{i=1}^{{n^{\prime }}_{11}}d_{11i}^{\prime }{y^{\prime }}_{11i}}{\sum_{i=1}^{{n^{\prime }}_{11}}d_{11i}^{\prime }{x^{\prime }}_{11i}}x_{1a}^{\prime }+\left(\frac{\sum_{i=1}^{{n^{\prime }}_{12r}}d_{11i}^{\prime }d_{12ri}^{\prime }{y^{\prime }}_{12ri}}{\sum_{i=1}^{{n^{\prime }}_{12r}}d_{11i}^{\prime }d_{12ri}^{\prime }{x^{\prime }}_{12ri}}\right)x_{2a}^{\prime }$$

**Case II:** If ${q^{\prime }}_{11i}=1$ and ${q^{\prime }}_{12i}=1$ are substituted in Equation (54) then we have the regression type estimator as Equation (56)

(56) $$T_{GREG,a}^{\prime *}=\sum_{i=1}^{n_{11}^{\prime }}d_{11i}^{\prime }y_{11i}^{\prime }+\sum_{i=1}^{n_{12r}^{\prime }}d_{11i}^{\prime }d_{12ri}^{\prime }y_{12ri}^{\prime }+\frac{\sum_{i=1}^{n_{11}^{\prime }}d_{11i}^{\prime }x_{11i}^{\prime }y_{11i}^{\prime }}{\sum_{i=1}^{n_{11}^{\prime }}d_{11i}^{\prime }x_{11i}^{\prime 2}}\left(x_{1a}^{\prime }-\sum_{i=1}^{n_{11}^{\prime }}d_{11i}^{\prime }x_{11i}^{\prime }\right)+\frac{\sum_{i=1}^{{n^{\prime }}_{12r}}d_{11i}^{\prime }d_{12ri}^{\prime }{x^{\prime }}_{12ri}{y^{\prime }}_{12ri}}{\sum_{i=1}^{{n^{\prime }}_{12r}}d_{11i}^{\prime }d_{12ri}^{\prime }x_{12ri}^{\prime 2}}\left(x_{2a}^{\prime }-\sum_{i=1}^{{n^{\prime }}_{12r}}d_{11i}^{\prime }d_{12ri}^{\prime }{x^{\prime }}_{12ri}\right)$$

Now, the new form of the regression estimator will be in Equation (57)

(57) $$T_{GREG,a}^{\prime *}=\sum_{i=1}^{{n^{\prime }}_{11}}d_{11i}^{\prime }{y^{\prime }}_{11i}+\sum_{i=1}^{{n^{\prime }}_{12r}}d_{11i}^{\prime }d_{12ri}^{\prime }{y^{\prime }}_{12ri}+{\hat{\alpha }}_{1}\left(x_{1a}^{\prime }-\sum_{i=1}^{{n^{\prime }}_{11}}d_{11i}^{\prime }{x^{\prime }}_{11i}\right)+{\hat{\alpha }}_{2}\left(x_{2a}^{\prime }-\sum_{i=1}^{{n^{\prime }}_{12r}}d_{11i}^{\prime }d_{12ri}^{\prime }{x^{\prime }}_{12ri}\right)$$

where $\alpha_{1}=\frac{\sum_{i=1}^{{n^{\prime }}_{11}}d_{11i}^{\prime }{x^{\prime }}_{11i}{y^{\prime }}_{11i}}{\sum_{i=1}^{{n^{\prime }}_{11}}d_{11i}^{\prime }x_{11i}^{\prime 2}}$ and $\alpha_{2}=\frac{\sum_{i=1}^{{n^{\prime }}_{12r}}d_{11i}^{\prime }d_{12ri}^{\prime }{x^{\prime }}_{12ri}{y^{\prime }}_{12ri}}{\sum_{i=1}^{{n^{\prime }}_{12r}}d_{11i}^{\prime }d_{12ri}^{\prime }x_{12ri}^{\prime 2}}$.

**Case III:** It put down ${q^{\prime }}_{11i}\neq 1$ and ${q^{\prime }}_{12i}\neq 1$ in Equation (54), and then it can't get the terms of $t_{11i}^{\prime }$ and $t_{12i}^{\prime }$ exactly. Hence, we can take the idea of function used under [31]. Then Equation (43) and Equation (44) can be written for the response weight function in Equation (58)

(58) $${v^{\prime }}_{11i}=d_{11i}^{\prime }\;\exp\left({l^{\prime }}_{1}{x^{\prime }}_{11i}\right)$$

where, explanation can be write in Equation (59) and Equation (60)

(59) $$\exp\left(l_{1}^{\prime }{x^{\prime }}_{11i}\right)=1+\frac{\left(l_{1}^{\prime }{x^{\prime }}_{11i}\right)}{1!}+\frac{{\left(l_{1}^{\prime }{x^{\prime }}_{11i}\right)}^{2}}{2!}+\frac{{\left(l_{1}^{\prime }{x^{\prime }}_{11i}\right)}^{3}}{3!}+...=1+l_{1}^{\prime }{x^{\prime }}_{11i}\left(1+\frac{\left(l_{1}^{\prime }{x^{\prime }}_{11i}\right)}{2!}+\frac{{\left(l_{1}^{\prime }{x^{\prime }}_{11i}\right)}^{2}}{3!}+...\right)=1+l_{1}^{\prime }{x^{\prime }}_{11i}{q^{\prime }}_{11i}\text{.}$$

The value,

(60) $${q^{\prime }}_{11i}=1+\frac{\left(l_{1}^{\prime }{x^{\prime }}_{11i}\right)}{2!}+\frac{{\left(l_{1}^{\prime }{x^{\prime }}_{11i}\right)}^{2}}{3!}+...$$

Also the non-response weight function will be like Equation (61)

(61) $${v^{\prime }}_{12i}=d_{11i}^{\prime }d_{12ri}^{\prime }\;\exp\left(l_{2}^{\prime }{x^{\prime }}_{12ri}\right)$$

where, expansion shows in Equation (62), and Equation (63)

(62) $$\exp\left(l_{2}^{\prime }{x^{\prime }}_{12ri}\right)=1+\frac{\left(l_{2}^{\prime }{x^{\prime }}_{12ri}\right)}{1!}+\frac{{\left(l_{2}^{\prime }{x^{\prime }}_{12ri}\right)}^{2}}{2!}+\frac{{\left(l_{2}^{\prime }{x^{\prime }}_{12ri}\right)}^{3}}{3!}+...=1+l_{2}^{\prime }{x^{\prime }}_{12ri}\left(1+\frac{\left(l_{2}^{\prime }{x^{\prime }}_{12ri}\right)}{2!}+\frac{{\left(l_{2}^{\prime }{x^{\prime }}_{12ri}\right)}^{2}}{3!}+...\right)=1+l_{2}^{\prime }{x^{\prime }}_{12ri}{q^{\prime }}_{12i}$$

and,

(63) $${q^{\prime }}_{12i}=1+\frac{\left(l_{2}^{\prime }{x^{\prime }}_{12ri}\right)}{2!}+\frac{{\left(l_{2}^{\prime }{x^{\prime }}_{12ri}\right)}^{2}}{3!}+...$$

The values of $l_{1}^{\prime }$ and $l_{2}^{\prime }$ can be estimated with the help of approximation method Newton-Raphson. If new weights ${v^{\prime }}_{11i}$ and ${v^{\prime }}_{12i}$ are substituted in Equation (43), it comes to be estimator like Exponential (Nguyen) in Equation (64)

(64) $${T_{NG}^{\prime *}}_{,a}=\sum_{i=1}^{{n^{\prime }}_{11}}{d^{\prime }}_{11i}{y^{\prime }}_{11i}\;\exp\left(l_{1}^{\prime }{x^{\prime }}_{11i}\right)+\sum_{i=1}^{{n^{\prime }}_{12r}}{d^{\prime }}_{11i}{d^{\prime }}_{12ri}{y^{\prime }}_{12ri}\;\exp\left(l_{2}^{\prime }{x^{\prime }}_{12ri}\right)\text{.}$$

In the real scenario many models can fit to be positively skew in nature. For example they are an exponential, power and others. The important real field situations are: income of households, or average income of families in a specific region. Hence, we are proposing a power based model. The power weight function of the response units can be written by Equation (65)

(65) $${u^{\prime }}_{11i}={d^{\prime }}_{11i}{\eta^{\prime }}_{1}^{{x^{\prime }}_{11i}}\text{.}$$

The justification of power is showing in Equation (66)

(66) $${\left({\eta^{\prime }}_{1}\right)}^{{x^{\prime }}_{11i}}=1+{\eta^{\prime }}_{1}{x^{\prime }}_{11i}\left\{-\left(\frac{1}{{\eta^{\prime }}_{1}}-1\right)+\frac{\left({x^{\prime }}_{11i}-1\right)\left(1-{\eta^{\prime }}_{1}\right)\left(\frac{1}{{\eta^{\prime }}_{1}}-1\right)}{2!}-\frac{\left({x^{\prime }}_{11i}-1\right)\left({x^{\prime }}_{11i}-2\right){\left(1-{\eta^{\prime }}_{1}\right)}^{2}\left(\frac{1}{{\eta^{\prime }}_{1}}-1\right)}{3!}+...\right\}$$

where, Equation (67) is the value of

(67) $${q^{\prime }}_{11i}=\left\{-\left(\frac{1}{{\eta^{\prime }}_{1}}-1\right)+\frac{\left({x^{\prime }}_{11i}-1\right)\left(1-{\eta^{\prime }}_{1}\right)\left(\frac{1}{{\eta^{\prime }}_{1}}-1\right)}{2!}-\frac{\left({x^{\prime }}_{11i}-1\right)\left({x^{\prime }}_{11i}-2\right){\left(1-{\eta^{\prime }}_{1}\right)}^{2}\left(\frac{1}{{\eta^{\prime }}_{1}}-1\right)}{3!}+...\right\}$$

The value of the respondent can written in the understandable form is in Equation (68)

(68) $${u^{\prime }}_{11i}={d^{\prime }}_{11i}\left(1+{\eta^{\prime }}_{1}{q^{\prime }}_{11i}{x^{\prime }}_{11i}\right)\text{.}$$

The non-response weight function is given by Equation (69)

(69) $${u^{\prime }}_{12ri}={d^{\prime }}_{11i}{d^{\prime }}_{12ri}{\eta^{\prime }}_{2}^{{x^{\prime }}_{12ri}}\text{.}$$

Its form can be written by Equation (70)

(70) $${\left({\eta^{\prime }}_{2}\right)}^{{x^{\prime }}_{12ri}}=1+{\eta^{\prime }}_{2}{x^{\prime }}_{12ri}\left\{-\left(\frac{1}{{\eta^{\prime }}_{2}}-1\right)+\frac{\left({x^{\prime }}_{12ri}-1\right)\left(1-{\eta^{\prime }}_{2}\right)\left(\frac{1}{{\eta^{\prime }}_{2}}-1\right)}{2!}-\frac{\left({x^{\prime }}_{12ri}-1\right)\left({x^{\prime }}_{12ri}-2\right){\left(1-{\eta^{\prime }}_{2}\right)}^{2}\left(\frac{1}{{\eta^{\prime }}_{2}}-1\right)}{3!}+...\right\}$$

where, Equation (71) is value of ${q^{\prime }}_{12i}$.

(71) $${q^{\prime }}_{12i}=\left\{-\left(\frac{1}{{\eta^{\prime }}_{2}}-1\right)+\frac{\left({x^{\prime }}_{12ri}-1\right)\left(1-{\eta^{\prime }}_{2}\right)\left(\frac{1}{{\eta^{\prime }}_{2}}-1\right)}{2!}-\frac{\left({x^{\prime }}_{12ri}-1\right)\left({x^{\prime }}_{12ri}-2\right){\left(1-{\eta^{\prime }}_{2}\right)}^{2}\left(\frac{1}{{\eta^{\prime }}_{2}}-1\right)}{3!}+...\right\}$$

The new first phase weight is written in Equation (72)

(72) $${u^{\prime }}_{12ri}={d^{\prime }}_{11i}{d^{\prime }}_{12ri}\left(1+{\eta^{\prime }}_{2}{q^{\prime }}_{12i}{x^{\prime }}_{12ri}\right)\text{.}$$

It is substituted in Equation (43), Equation (73) found the power function based estimator

(73) $${T_{Power}^{\prime *}}_{,a}=\sum_{i=1}^{{n^{\prime }}_{11i}}{d^{\prime }}_{11i}{y^{\prime }}_{11i}{\eta^{\prime }}_{1}^{{x^{\prime }}_{11i}}+\sum_{i=1}^{{n^{\prime }}_{12r}}{d^{\prime }}_{11i}{d^{\prime }}_{12ri}{y^{\prime }}_{12ri}{\eta^{\prime }}_{2}^{{x^{\prime }}_{12ri}}\text{.}$$

In the two phase sampling x is used for ath domain total. Second phase sample was taken from the first phase sample. Both Equation (74) and Equation (75) show response and non-response total values of helping variable are as

(74) $$\sum_{i=1}^{n_{11}^{\prime \prime }}t_{11i}^{\prime \prime *}x_{11i}^{\prime \prime }=x_{1a}^{\prime }$$

and

(75) $$\sum_{i=1}^{n_{12r}^{\prime \prime }}t_{12i}^{\prime \prime *}x_{12ri}^{\prime \prime }=x_{2a}^{\prime }\;\text{.}$$

We are considering minimum chi-square distance function again under remark 1 as Equation (76)

(76) $$D_{2}\left(t_{11i}^{\prime \prime *},{d^{\prime \prime }}_{11i}\right)=\sum_{i=1}^{n_{11}^{\prime \prime }}\frac{{\left(t_{11i}^{\prime \prime *}-{t_{11i}^{\prime *}d}_{11i}^{\prime \prime }\right)}^{2}}{2d_{11i}^{\prime \prime }{t_{11i}^{\prime *}q^{\prime \prime }}_{11i}}+\sum_{i=1}^{n_{12r}^{\prime \prime }}\frac{{\left(t_{12i}^{\prime \prime *}-{{t_{11i}^{\prime *}d}_{11i}^{\prime \prime }d}_{12ri}^{\prime \prime }\right)}^{2}}{2{t_{11i}^{\prime *}d}_{11i}^{\prime \prime }d_{12ri}^{\prime \prime }{q^{\prime \prime }}_{12i}}\text{.}$$

Now, the Lagrange's function is written as Equation (77)

(77) $$L\left(t_{11i}^{\prime \prime *},t_{12i}^{\prime \prime *},\varsigma_{3},\varsigma_{4}\right)=\frac{1}{2}\sum_{i=1}^{n_{11}^{\prime \prime }}\frac{{\left(t_{11i}^{\prime \prime *}-{t_{11i}^{\prime *}d}_{11i}^{\prime \prime }\right)}^{2}}{{t_{11i}^{\prime *}d}_{11i}^{\prime \prime }q_{11i}^{\prime \prime }}+\varsigma_{3}\left(x_{1a}^{\prime }-\sum_{i=1}^{n_{11}^{\prime \prime }}t_{11i}^{\prime \prime *}x_{11i}^{\prime \prime }\right)+\frac{1}{2}\sum_{i=1}^{n_{12r}^{\prime \prime }}\frac{{\left(t_{12i}^{\prime \prime *}-{t_{11i}^{\prime *}d}_{11i}^{\prime \prime }d_{12ri}^{\prime \prime }\right)}^{2}}{2t_{11i}^{\prime *}d_{11i}^{\prime \prime }d_{12ri}^{\prime \prime }q_{12ri}^{\prime \prime }}+\varsigma_{4}\left(x_{2a}^{\prime }-\sum_{i=1}^{n_{12r}^{\prime \prime }}{t_{11i}^{\prime *}t}_{12i}^{\prime \prime *}x_{12ri}^{\prime \prime *}\right)\text{.}$$

where $\varsigma_{3}$ and $\varsigma_{4}$ are the response and non-response Lagrange's multipliers. We are interested in getting the value of new weights $t_{11i}^{\prime \prime *}$ and $t_{12i}^{\prime \prime *}$. The partially differentiating Equation (77) w.r.to two weights $t_{11i}^{\prime \prime *}$, $t_{12i}^{\prime \prime *}$ and equates to $\frac{\partial L\left(t_{11i}^{\prime \prime *},t_{12i}^{\prime \prime *},{\;\varsigma }_{3},{\;\varsigma }_{4}\right)}{\partial L\left(t_{11i}^{\prime \prime *}\right)}=0$ and $\frac{\partial L\left(t_{11i}^{\prime \prime *},{\;t}_{12i}^{\prime \prime *},{\;\varsigma }_{3},{\;\varsigma }_{4}\right)}{\partial L\left(t_{12i}^{\prime \prime *}\right)}=0$. We obtain response weight as given in Equation (78)

(78) $$t_{11i}^{\prime \prime *}={t_{11i}^{\prime *}d}_{11i}^{\prime \prime *}\left(1+\varsigma_{3}{q^{\prime \prime }}_{11i}{x^{\prime \prime }}_{11i}\right)$$

and non-response weight in Equation (79)

(79) $$t_{12i}^{\prime \prime *}={t_{11i}^{\prime *}d_{11i}^{\prime \prime }d}_{12ri}^{\prime \prime }\left(1+\varsigma_{4}{q^{\prime \prime }}_{12i}{x^{\prime \prime }}_{12ri}\right)\text{.}$$

Both weights $t_{11i}^{\prime \prime *}$ and $t_{12i}^{\prime \prime *}$ are putting in Equation (74) and Equation (75), respectively, both Equation (80) and Equation (81) show both $\varsigma_{3}$ and $\varsigma_{4}$.

(80) $$\varsigma_{3}=\frac{\left(x_{1a}^{\prime }-\sum_{i=1}^{{n^{\prime \prime }}_{11}}{t_{11i}^{\prime *}d}_{11i}^{\prime \prime }{x^{\prime \prime }}_{11i}\right)}{\sum_{i=1}^{{n^{\prime \prime }}_{11}}t_{11i}^{\prime *}d_{11i}^{\prime \prime }{q^{\prime \prime }}_{11i}x_{11i}^{\prime \prime 2}}$$

and

(81) $$\varsigma_{4}=\frac{\left(x_{2a}^{\prime }-\sum_{i=1}^{{n^{\prime \prime }}_{12r}}{t_{11i}^{\prime *}d}_{11i}^{\prime \prime }d_{12ri}^{\prime \prime }{x^{\prime \prime }}_{12ri}\right)}{\sum_{i=1}^{{n^{\prime \prime }}_{12r}}{t_{11i}^{\prime *}d}_{11i}^{\prime \prime }d_{12ri}^{\prime \prime }{q^{\prime \prime }}_{12i}x_{12ri}^{\prime \prime 2}}\text{.}$$

The second phase Lagrange's multiplier's values of $\varsigma_{3}$ and $\varsigma_{4}$ are substituted in Equation (78) and Equation (79), the respondent weight in Equation (82) and non-respondent weights in Equation (83) are given by

(82) $$t_{11i}^{\prime \prime *}=t_{11i}^{\prime *}d_{11i}^{\prime \prime }\left(1+\frac{\left(x_{1a}^{\prime }-\sum_{i=1}^{{n^{\prime \prime }}_{11}}{t_{11i}^{\prime *}d}_{11i}^{\prime \prime }{x^{\prime \prime }}_{11i}\right)}{\sum_{i=1}^{{n^{\prime \prime }}_{11}}{t_{11i}^{\prime *}d}_{11i}^{\prime \prime }{q^{\prime \prime }}_{11i}{x^{\prime \prime }}_{11i}^{2}}{q^{\prime \prime }}_{11i}{x^{\prime \prime }}_{11i}\right)$$

and

(83) $$t_{12i}^{\prime \prime *}={t_{11i}^{\prime *}d}_{11i}^{\prime \prime }d_{12ri}^{\prime \prime }\left(1+\frac{\left(x_{2a}^{\prime \prime }-\sum_{i=1}^{{n^{\prime \prime }}_{12r}}d_{11i}^{\prime \prime }d_{12ri}^{\prime \prime }{x^{\prime \prime }}_{12ri}\right)}{\sum_{i=1}^{{n^{\prime \prime }}_{12r}}{t_{11i}^{\prime *}d}_{11i}^{\prime \prime }d_{12ri}^{\prime \prime }{q^{\prime \prime }}_{12i}x_{12ri}^{\prime \prime 2}}{q^{\prime \prime }}_{12i}{x^{\prime \prime }}_{12ri}\right)\text{.}$$

Further, it is putting down both new weights ${t^{\prime }}_{11i}^{\prime *}$ and $t_{12i}^{\prime \prime *}$ put in Equation (74). The proposed estimator at second phase given in Equation (84)

(84) $${T^{\prime \prime }}_{P,a}^{*}=\sum_{i=1}^{n_{11}^{\prime \prime }}d_{11i}^{\prime \prime }y_{11i}^{\prime \prime }+\sum_{i=1}^{n_{12r}^{\prime \prime }}d_{12ri}^{\prime \prime }y_{12ri}^{\prime \prime }+\left(\frac{\sum_{i=1}^{n_{11}^{\prime \prime }}{t_{11i}^{\prime *}d}_{11i}^{\prime \prime }q_{11i}^{\prime \prime }x_{11i}^{\prime \prime }y_{11i}^{\prime \prime }}{\sum_{i=1}^{n_{11}^{\prime \prime }}{t_{11i}^{\prime *}d}_{11i}^{\prime \prime }q_{11i}^{\prime \prime }{x^{\prime \prime }}_{11i}^{2}}\right)\left(x_{1a}^{\prime }-\sum_{i=1}^{n_{11}^{\prime \prime }}{t_{11i}^{\prime *}d}_{11i}^{\prime }x_{11i}^{\prime }\right)+\left(\frac{\sum_{i=1}^{{n^{\prime \prime }}_{12r}}{t_{11i}^{\prime *}d}_{11i}^{\prime \prime }d_{12ri}^{\prime \prime }{q^{\prime \prime }}_{12i}{x^{\prime \prime }}_{12ri}{y^{\prime \prime }}_{12ri}}{\sum_{i=1}^{{n^{\prime \prime }}_{12r}}{t_{11i}^{\prime *}d}_{11i}^{\prime \prime }d_{12ri}^{\prime \prime }{q^{\prime \prime }}_{12i}{x^{\prime \prime }}_{12ri}^{2}}\right)\left(x_{2a}^{\prime }-\sum_{i=1}^{{n^{\prime \prime }}_{12r}}{t_{11i}^{\prime *}d}_{11i}^{\prime \prime }d_{12ri}^{\prime \prime }{x^{\prime \prime }}_{12ri}\right)\text{.}$$

We are giving some special cases of Equation (84).

**Case I:** If q″11i=1x″11i and q″12i=1x″12ri are put down in Equation (84). The equation reduces to ratio type estimator which is written in Equation (85)

(85) $$T_{Ratio,a}^{\prime \prime *}=\frac{\sum_{i=1}^{{n^{\prime \prime }}_{11}}{t_{11i}^{\prime *}d}_{11i}^{\prime \prime }{y^{\prime \prime }}_{11i}}{\sum_{i=1}^{{n^{\prime \prime }}_{11}}{t_{11i}^{\prime *}d}_{11i}^{\prime \prime }{x^{\prime \prime }}_{11i}}x_{1a}^{\prime }+\left(\frac{\sum_{i=1}^{{n^{\prime \prime }}_{12r}}{t_{11i}^{\prime *}d}_{11i}^{\prime \prime }d_{12ri}^{\prime \prime }{y^{\prime \prime }}_{12ri}}{\sum_{i=1}^{{n^{\prime \prime }}_{12r}}{t_{11i}^{\prime *}d}_{11i}^{\prime \prime }d_{12ri}^{\prime \prime }{x^{\prime \prime }}_{12ri}}\right)x_{2a}^{\prime }$$

**Case II:** If, we are substituted ${q^{\prime \prime }}_{11i}=1$ and ${q^{\prime \prime }}_{12i}=1$ in Equation (84), the proposed estimator reduces to be regression type estimator like in Equation (86)

(86) $$T_{GREG,a}^{\prime \prime *}=\sum_{i=1}^{n_{11}^{\prime \prime }}d_{11i}^{\prime \prime }y_{11i}^{\prime \prime }+\sum_{i=1}^{n_{12r}^{\prime \prime }}d_{11i}^{\prime \prime }d_{12ri}^{\prime \prime }y_{12ri}^{\prime \prime }+\frac{\sum_{i=1}^{n_{11}^{\prime \prime }}{t_{11i}^{\prime *}d}_{11i}^{\prime \prime }x_{11i}^{\prime \prime }y_{11i}^{\prime \prime }}{\sum_{i=1}^{n_{11}^{\prime \prime }}{t_{11i}^{\prime *}d}_{11i}^{\prime \prime }x_{11i}^{\prime \prime 2}}\left(x_{1a}^{\prime }-\sum_{i=1}^{n_{11}^{\prime \prime }}{t_{11i}^{\prime *}d}_{11i}^{\prime \prime }x_{11i}^{\prime \prime }\right)+\frac{\sum_{i=1}^{{n^{\prime \prime }}_{12r}}{t_{12i}^{\prime *}d}_{11i}^{\prime \prime }d_{12ri}^{\prime \prime }{x^{\prime \prime }}_{12ri}{y^{\prime \prime }}_{12ri}}{\sum_{i=1}^{{n^{\prime \prime }}_{12r}}{t_{12i}^{\prime *}d}_{11i}^{\prime \prime }d_{12ri}^{\prime \prime }x_{12ri}^{\prime \prime 2}}\left(x_{2a}^{\prime }-\sum_{i=1}^{{n^{\prime \prime }}_{12r}}t_{12i}^{\prime *}d_{11i}^{\prime \prime }d_{12ri}^{\prime \prime }{x^{\prime \prime }}_{12ri}\right)\text{.}$$

The new form of the regression estimator will be in Equation (87)

(87) $$T_{GREG,a}^{\prime \prime *}=\sum_{i=1}^{{n^{\prime \prime }}_{11}}t_{11i}^{\prime *}d_{11i}^{\prime \prime }{y^{\prime \prime }}_{11i}+\sum_{i=1}^{{n^{\prime \prime }}_{12r}}{t_{12i}^{\prime *}d}_{11i}^{\prime \prime }d_{12ri}^{\prime \prime }{y^{\prime \prime }}_{12ri}+{\hat{\alpha }}_{1}\left(x_{1a}^{\prime }-\sum_{i=1}^{{n^{\prime \prime }}_{11}}{t_{11i}^{\prime *}d}_{11i}^{\prime \prime }{x^{\prime \prime }}_{11i}\right)+{\hat{\alpha }}_{2}\left(x_{2a}^{\prime }-\sum_{i=1}^{{n^{\prime \prime }}_{12r}}{t_{12i}^{\prime *}d}_{11i}^{\prime \prime }d_{12ri}^{\prime \prime }{x^{\prime \prime }}_{12ri}\right)$$

where ${\hat{\alpha }}_{1}=\frac{\sum_{i=1}^{{n^{\prime \prime }}_{11}}{t_{11i}^{\prime *}d}_{11i}^{\prime \prime }{x^{\prime \prime }}_{11i}{y^{\prime \prime }}_{11i}}{\sum_{i=1}^{{n^{\prime \prime }}_{11}}{t_{11i}^{\prime *}d}_{11i}^{\prime \prime }x_{11i}^{\prime \prime 2}}$ and ${\hat{\alpha }}_{2}=\frac{\sum_{i=1}^{{n^{\prime \prime }}_{12r}}{t_{12i}^{\prime *}d}_{11i}^{\prime \prime }d_{12ri}^{\prime \prime }{x^{\prime \prime }}_{12ri}{y^{\prime \prime }}_{12ri}}{\sum_{i=1}^{{n^{\prime \prime }}_{12r}}{t_{12i}^{\prime *}d_{11i}^{\prime \prime }d}_{12ri}^{\prime \prime }x_{12ri}^{\prime \prime 2}}$.

**Case III:** If ${q^{\prime \prime }}_{11i}\neq 1$ and ${q^{\prime \prime }}_{12i}\neq 1$ are substituted in Equations (74), (75), they can't separate completely with respect to of $t_{11i}^{\prime \prime }$ and $t_{12i}^{\prime \prime }$. Therefore, it can take the functional form based under [31] form. Using Equation (78) and Equation (79), the respondent weight function can be formulated as follows in Equation (88)

(88) $${v^{\prime \prime }}_{11i}=t_{11i}^{\prime \prime }d_{11i}^{\prime \prime }\;\exp\left({l^{\prime \prime }}_{1}{x^{\prime \prime }}_{11i}\right)$$

where, both Equation (89) and Equation (90) derive exponential weight

(89) $$\exp\left(l_{1}^{\prime \prime }{x^{\prime \prime }}_{11i}\right)=1+\frac{\left(l_{1}^{\prime \prime }{x^{\prime \prime }}_{11i}\right)}{1!}+\frac{{\left(l_{1}^{\prime \prime }{x^{\prime \prime }}_{11i}\right)}^{2}}{2!}+\frac{{\left(l_{1}^{\prime \prime }{x^{\prime \prime }}_{11i}\right)}^{3}}{3!}+...=1+l_{1}^{\prime \prime }{x^{\prime \prime }}_{11i}\left(1+\frac{\left(l_{1}^{\prime \prime }{x^{\prime \prime }}_{11i}\right)}{2!}+\frac{{\left(l_{1}^{\prime \prime }{x^{\prime \prime }}_{11i}\right)}^{2}}{3!}+...\right)=1+l_{1}^{\prime \prime }{x^{\prime \prime }}_{11i}{q^{\prime \prime }}_{11i}$$

and the value,

(90) $${q^{\prime }}_{11i}=1+\frac{\left(l_{1}^{\prime \prime }{x^{\prime \prime }}_{11i}\right)}{2!}+\frac{{\left(l_{1}^{\prime \prime }{x^{\prime \prime }}_{11i}\right)}^{2}}{3!}+...$$

where, the non-respondent weight function given by Equation (91)

(91) $${v^{\prime \prime }}_{12i}=d_{11i}^{\prime \prime }d_{12i}^{\prime \prime }\;\exp\left(l_{2}^{\prime \prime }{x^{\prime \prime }}_{12ri}\right)\text{.}$$

The calibration weight on the second phase in Equation (92) and chosen constant in Equation (93) can be written by

(92) $$\exp\left(l_{2}^{\prime \prime }{x^{\prime \prime }}_{12ri}\right)=1+\frac{\left(l_{2}^{\prime \prime }{x^{\prime \prime }}_{12ri}\right)}{1!}+\frac{{\left(l_{2}^{\prime \prime }{x^{\prime \prime }}_{12ri}\right)}^{2}}{2!}+\frac{{\left(l_{2}^{\prime \prime }{x^{\prime \prime }}_{12ri}\right)}^{3}}{3!}+...=1+l_{2}^{\prime \prime }{x^{\prime \prime }}_{12ri}\left(1+\frac{\left(l_{2}^{\prime \prime }{x^{\prime \prime }}_{12ri}\right)}{2!}+\frac{{\left(l_{2}^{\prime \prime }{x^{\prime \prime }}_{12ri}\right)}^{2}}{3!}+...\right)=1+l_{2}^{\prime \prime }{x^{\prime \prime }}_{12ri}{q^{\prime }}_{12i}$$

(93) $$\text{where},{q^{\prime \prime }}_{12i}=1+\frac{\left(l_{2}^{\prime \prime }{x^{\prime \prime }}_{12ri}\right)}{2!}+\frac{{\left(l_{2}^{\prime \prime }{x^{\prime \prime }}_{12ri}\right)}^{2}}{3!}+...$$

The values of $l_{1}^{\prime \prime }$ and $l_{2}^{\prime \prime }$ are estimated with the help of the Newton-Raphson. If it's estimated value ${v^{\prime \prime }}_{11i}$ and ${v^{\prime \prime }}_{12i}$ substituted in Equation (43), it comes to be the Nguyen form of the estimator as in Equation (94)

(94) $${T_{NG}^{\prime \prime *}}_{,a}=\sum_{i=1}^{{n^{\prime \prime }}_{11}}{d^{\prime \prime }}_{11i}{y^{\prime \prime }}_{11i}\;\exp\left(l_{1}^{\prime \prime }{x^{\prime \prime }}_{11i}\right)+\sum_{i=1}^{{n^{\prime \prime }}_{12r}}{d^{\prime \prime }}_{11i}{d^{\prime \prime }}_{12ri}{y^{\prime \prime }}_{12ri}\;\exp\left(l_{2}^{\prime \prime }{x^{\prime \prime }}_{12ri}\right)\text{.}$$

Further, the power weight function in Equation (95) of the respondent unit is given by

(95) $${u^{\prime \prime }}_{11i}={d^{\prime \prime }}_{11i}{{\eta^{\prime \prime }}_{1}}^{{x^{\prime \prime }}_{11i}}\text{.}$$

It is written in Equation (96) by

(96) $${\left({\eta^{\prime \prime }}_{1}\right)}^{{x^{\prime \prime }}_{11i}}=1+{\eta^{\prime \prime }}_{1}{x^{\prime \prime }}_{11i}\left\{-\left(\frac{1}{{\eta^{\prime \prime }}_{1}}-1\right)+\frac{\left({x^{\prime \prime }}_{11i}-1\right)\left(1-{\eta^{\prime \prime }}_{1}\right)\left(\frac{1}{{\eta^{\prime \prime }}_{1}}-1\right)}{2!}-\frac{\left({x^{\prime \prime }}_{11i}-1\right)\left({x^{\prime \prime }}_{11i}-2\right){\left(1-{\eta^{\prime \prime }}_{1}\right)}^{2}\left(\frac{1}{{\eta^{\prime \prime }}_{1}}-1\right)}{3!}+...\right\}$$

where, the ${q^{\prime \prime }}_{11i}$ value given in Equation (97)

(97) $${q^{\prime \prime }}_{11i}=\left\{-\left(\frac{1}{{\eta^{\prime \prime }}_{1}}-1\right)+\frac{\left({x^{\prime \prime }}_{11i}-1\right)\left(1-{\eta^{\prime \prime }}_{1}\right)\left(\frac{1}{{\eta^{\prime \prime }}_{1}}-1\right)}{2!}-\frac{\left({x^{\prime \prime }}_{11i}-1\right)\left({x^{\prime \prime }}_{11i}-2\right){\left(1-{\eta^{\prime \prime }}_{1}\right)}^{2}\left(\frac{1}{{\eta^{\prime \prime }}_{1}}-1\right)}{3!}+...\right\}$$

The simple form of the non-respondent weight in Equation (98) can be

(98) $${u^{\prime \prime }}_{11i}={d^{\prime \prime }}_{11i}\left(1+{\eta^{\prime \prime }}_{1}{q^{\prime \prime }}_{11i}{x^{\prime \prime }}_{11i}\right)\text{.}$$

The non-respondent weight function in Equation (99) is given by

(99) $${u^{\prime \prime }}_{12ri}={d^{\prime \prime }}_{11i}{d^{\prime \prime }}_{12ri}{{\eta^{\prime \prime }}_{2}}^{{x^{\prime \prime }}_{12ri}}\text{.}$$

It can be written as given in Equation (100)

(100) $${\left({\eta^{\prime \prime }}_{2}\right)}^{{x^{\prime \prime }}_{12ri}}=1++{\eta^{\prime \prime }}_{2}{x^{\prime \prime }}_{12ri}\left\{-\left(\frac{1}{{\eta^{\prime \prime }}_{2}}-1\right)+\frac{\left({x^{\prime \prime }}_{12ri}-1\right)\left(1-{\eta^{\prime \prime }}_{2}\right)\left(\frac{1}{{\eta^{\prime \prime }}_{2}}-1\right)}{2!}-\frac{\left({x^{\prime \prime }}_{12ri}-1\right)\left({x^{\prime \prime }}_{12ri}-2\right){\left(1-{\eta^{\prime \prime }}_{2}\right)}^{2}\left(\frac{1}{{\eta^{\prime \prime }}_{2}}-1\right)}{3!}+...\right\}$$

where, the value of ${q^{\prime \prime }}_{12i}$ is written in Equation (101)

(101) $${q^{\prime \prime }}_{12i}=\left\{-\left(\frac{1}{{\eta^{\prime \prime }}_{2}}-1\right)+\frac{\left({x^{\prime \prime }}_{12ri}-1\right)\left(1-{\eta^{\prime \prime }}_{2}\right)\left(\frac{1}{{\eta^{\prime \prime }}_{2}}-1\right)}{2!}-\frac{\left({x^{\prime \prime }}_{12ri}-1\right)\left({x^{\prime \prime }}_{12ri}-2\right){\left(1-{\eta^{\prime \prime }}_{2}\right)}^{2}\left(\frac{1}{{\eta^{\prime \prime }}_{2}}-1\right)}{3!}+...\right\}$$

The second phase weight is discussed in Equation (102)

(102) $${u^{\prime \prime }}_{12ri}={d^{\prime \prime }}_{11i}{d^{\prime \prime }}_{12ri}\left(1+{\eta^{\prime \prime }}_{2}{q^{\prime \prime }}_{12i}{x^{\prime \prime }}_{12ri}\right)\text{.}$$

It is substituted in Equation (43), with the help of remark 1 and remark 2. We obtain the proposed power function which is in Equation (103)

(103) $${T_{Power}^{\prime \prime *}}_{,a}=\sum_{i=1}^{{n^{\prime \prime }}_{11i}}{d^{\prime \prime }}_{11i}{y^{\prime \prime }}_{11i}{{\eta^{\prime \prime }}_{1}}^{{x^{\prime \prime }}_{11i}}+\sum_{i=1}^{{n^{\prime \prime }}_{12r}}{d^{\prime \prime }}_{11i}{d^{\prime \prime }}_{12ri}{y^{\prime \prime }}_{12ri}{{\eta^{\prime \prime }}_{2}}^{{x^{\prime \prime }}_{12ri}}\text{.}$$

We can write expectation of the power based estimator

$$E\left({T_{Power}^{\prime \prime *}}_{,a}\right)\neq Y_{a}\text{.}$$

This suggests that the power type estimator is not an unbiased estimator for entire domain. It implies that as sample size increases and approaches a large sample, the estimators of the model-based approach converge tend towards the estimators of the design approach. This convergence suggests that the model estimators become more similar to the estimators derived from the design-based approach as sample size increases. The proposed power based approach introduces bias that arises due to specific characteristics and assumptions of the different trills and accessed their efficiency in estimating the desired parameters. A simulation studies provide available tool for understanding the behaviour and properties of the estimators in practical setup. Specifically, theoretically analysis may be challenging or inconclusive.

## 6 Simulation study using two phase sampling

The same data has been used from the Swedish community [33] for empirical evaluation. It considers only six areas 1, 2, 3, 4, 5, and 6 with sizes of 48, 32, 38, 41, 15, and 29, respectively. The limitation is that the supporting information of the domains is not available, but the response and nonresponse information should be known in advance. The sample of the first phase is drawn about 60 percentage from each area. The remaining procedures are similar to those used in the first part of the study in this manuscript. In this situation 30, 40 and 50 sizes sample my not take due to not sufficient non-respondent are in the domains. The number of times the process is repeated to 6000 times. To check the performance of the indirect estimators the values for ARB and SRSE are calculated in Table 4.

**Table 4:** The value of ARB and SRSE for indirect estimators using minimum chi-square distance having regression, ratio, Nguyen and power type estimatorsin the presence of 30% non-response for the domains 1, 2, 3, 4, 5 and 6.

Sample Size	Estimators	Domains					
		1	2	3	4	5	6
		n_2r_ = 12					
	$T_{GREG,a}^{\prime \prime *}$	78.23 (29.14)	162.41 (83.42)	152.39 (74.23)	143.35 (30.42)	169.34 (40.13)	142.61 (48.78)
	$T_{Ratio,a}^{\prime \prime *}$	41.45 (28.36)	32.67 (7.83)	73.78 (6.58)	34.46 (8.32)	69.17 (30.57)	35.29 (8.77)
	$T_{NG,a}^{\prime \prime *}$	36.34 (24.81)	28.72 (7.05)	65.57 (5.38)	30.61 (6.76)	37.06 (26.39)	31.37 (6.45)
	$T_{Power,a}^{\prime \prime *}$	32.72 (20.37)	26.58 (6.23)	58.26 (4.61)	27.89 (5.69)	34.17 (23.21)	27.63 (4.84)
		n_2r_ = 15					
	$T_{GREG,a}^{\prime \prime *}$	72.36 (27.25)	136.24 (78.23)	146.48 (69.79)	132.58 (27.74)	152.67 (38.89)	128.53 41.47
	$T_{Ratio,a}^{\prime \prime *}$	36.41 (26.41)	28.86 (6.78)	67.37 (6.21)	31.43 (7.68)	47.36 (28.43)	33.51 (8.01)
	$T_{NG,a}^{\prime \prime *}$	33.56 (22.14)	26.34 (5.68)	62.53 (5.36)	27.31 (6.69)	42.57 (24.28)	29.62 (6.21)
	$T_{Power,a}^{\prime \prime *}$	30.13 (18.71)	24.23 (4.18)	56.24 (4.35)	25.34 (5.46)	32.31 (22.13)	27.29 (4.38)
90	n_2r_ = 14						
	$T_{GREG,a}^{\prime \prime *}$	77.15 (26.11)	149.23 (72.73)	149.57 (67.52)	139.76 (25.37)	159.42 (38.21)	138.21 (40.15)
	$T_{Ratio,a}^{\prime \prime *}$	38.57 (25.67)	29.31 (6.47)	67.42 (5.45)	29.35 (7.85)	60.21 (27.13)	34.31 (7.27)
	$T_{NG,a}^{\prime \prime *}$	32.34 (21.45)	25.71 (5.37)	58.78 (5.05)	27.62 (6.97)	54.35 (21.23)	27.16 (6.21)
	$T_{Power,a}^{\prime \prime *}$	31.03 (12.24)	23.17 (4.89)	50.56 (4.21)	25.97 (5.93)	57.27 (18.31)	25.56 (3.93)
	n_2r_ = 19						
	$T_{GREG,a}^{\prime \prime *}$	71.63 (26.94)	129.56 (69.21)	128.26 (64.87)	118.42 (23.92)	131.67 (36.89)	119.23 32.73
	$T_{Ratio,a}^{\prime \prime *}$	32.35 (23.43)	28.04 (6.07)	55.95 (5.07)	27.92 (6.93)	56.09 (25.52)	31.72 (7.01)
	$T_{NG,a}^{\prime \prime *}$	29.21 (20.16)	23.37 (5.17)	49.58 (4.56)	25.78 (5.25)	49.23 (19.62)	24.23 (5.74)
	$T_{Power,a}^{\prime \prime *}$	27.10 (11.92)	21.25 (4.32)	45.23 (4.04)	24.36 (4.96)	45.41 (16.23)	22.73 (3.47)

(): represent absolute relative bias of the estimators.

On the basis of information provided by Table 4, it suggests that the study evaluate for different domains for indirect generalized regression, ratio, Nguyen and power types estimator. The evaluation was conducted using the minimum chi-square distance approach in the presence of 30 percentage nonresponse for the domains 1, 2, 3, 4, 5 and 6. The study obtained that the two phase sample is utilized a power-based estimator outperformed other estimators for domains. This indicates thatit is incorporating two phase sample approach along with two phase sampling approach. The power based estimator yielded more accurate and precise results as compared to other estimators given in the domain. The estimation of the nonresponse was influenced by the choice of estimators in different sampling sizes. As the sample size increased simulate relative standard error decreased and indicating improved estimators precision. It is also seen that subsample from the non-response is different for both sizes 70 and 90. The performance of the method is also constituted by the first phase sample information. The efficiency of the estimators is also different. The estimated value of SRSE for domain 5 is the largest among all domains. The estimator GREG performs poorly among all the estimators in the domain in terms of SRSE. The power based estimator was performed better than GREG, ratio, and Nguyen estimators for all domains. Comparing with four estimators with and without two-phase sampling, the estimator without two-phase sampling performed better than one with two phase sampling elaborated in Table 2, Table 4.

## 7 Recommendations

The extensive simulation has concluded in this study. It has provided empirical evidence supporting total estimation. It demonstrated the effectiveness and usefulness of indirect estimators. These estimators have also been employed to address the challenge of a small number of available units within the study domain. We are focusing solely on examining the domain. It accounts the importance of the surrounding units. By considering units information, the performance of the power based estimation was shown to be improved, particularly in our crucial finding of the study is that the presence of unit non-response. Indirect power type estimator is outperformed to other estimators such as indirect ratio, generalized regression and Nguyen estimators for Swedish community data. This highlights the superiority of the power based estimator in the situations where the units of the target variable are not

available which is common in both public and private sectors. Furthermore, the present study has emphasized in many cases when the availability of the suitable socioeconomic variables are limited in low and middle-income countries. In these cases the use of indirect estimator indirect estimator becomes crucial due to small sample sizes and lack of information in local areas. Overall, the study finding supports the importance and effectiveness of indirect estimators. Particularly, proposed power based estimator. In the situations, if targets variable units are not easily access. These results may be implying for research and application in real field.

## 8 Applications

Indeed, the development of positively biased models having similar characteristic. It can be used when supporting variable units are missing. The application of such models can be particularly beneficial invarious domains including incomeareas, environment issues and epidemiological problems. By developing innovative methods, it accounts for missing data and incorporate positively biased. This in turn, can contribute to more informed decision-making, policy development, and understanding of complex social, environmental and health issues. Some other positive skewed models can be developed that have the same nature. It may use when the study variable is missing. It can also used for two phase sampling method. It can be applied in low income group areas, environmental issues and epidemiological issues through multi-auxiliary variables. The proposed work has wide applications in the real problems in the future research for survey statistician.

## Data availability statement

Data will be made available on request.

## CRediT authorship contribution statement

**Ashutosh Ashutosh:** Supervision, Methodology. **Marius Stefan:** Writing – review & editing. **Piyush Kant Rai:** Conceptualization. **Walid Emam:** Project administration, Funding acquisition. **Soofia Iftikhar:** Investigation, Data curation. **Malik Muhammad Anas:** Writing – review & editing, Visualization, Validation.

## Declaration of competing interest

The authors declare that they have no known competing financial interests or personal relationships that could have appeared to influence the work reported in this paper.
